# The referral trajectories for young people with depression in low-resource settings: A qualitative study from Ghana and Zimbabwe

**DOI:** 10.1371/journal.pone.0358255

**Published:** 2026-10-05

**Authors:** Precious Makiyi, Rufaro Mushonga, Rebecca Jopling, Raymond Akawire Aborigo, Franklin Glozah, Kimberley Goldsmith, Benedict Weobong, Dixon Chibanda, Melanie Abas, James January, Moses Kumwenda

**Affiliations:** 1 Department of Psychiatry & Mental Health, Kamuzu University of Health Sciences, Blantyre, Malawi; 2 Herse-Nord TB Initiative (HNTI), Kamuzu University of Health Sciences, Blantyre, Malawi; 3 Institute of Psychiatry, Psychology & Neurology, King’s College London, London, England; 4 Department of Global Public Health and Family Medicine, Faculty of Medicine and Health Sciences, University of Zimbabwe, Harare, Zimbabwe; 5 Malawi Liverpool-Welcome Trust, Kamuzu University of Health Sciences, Blantyre, Malawi; University of Education Winneba Faculty of Science Education, GHANA

## Abstract

**Background:**

Unlike in psychiatric disorders such as psychosis where treatment trajectories are well defined, little is known about pathways to care for depression which is increasingly prevalent among young people across the globe. Whilst empirical evidence is slowly becoming available on pathways to care for common mental disorders in the general population in Ghana and Zimbabwe, there is no study that specifically considered treatment pathways for young people.

**Objectives:**

This study explored trajectories used by young people (15–24 years) to access specialised mental healthcare in order to determine referral pathways for depression in Ghana and Zimbabwe.

**Methodology:**

This sub-analysis forms part of a larger formative, exploratory, descriptive qualitative study conducted within the Youth in Mind consortium. The study engaged 172 participants including young people, healthcare providers, policymakers, faith-based and traditional healers, lay health workers, and school staff across Ghana (N = 105) and Zimbabwe (N = 67) who were sampled purposively and using snowball techniques until saturation was reached. Data were analysed using an iterative combination of inductive and deductive coding, followed by thematic analysis.

**Results:**

Diagnostic and treatment trajectory for young people's access to specialized mental healthcare are complex in both Ghana and Zimbabwe and punctuated with several barriers that culminate in delayed access to treatments. Chief among the barriers facing young people are the catastrophic costs linked with accessing specialized mental health care, lack of autonomy of the youth, long waiting times perpetuated by a limited number of specialists and stigma. Opportunities for optimising access included the use of referral cards, which sped up the referral process, improved communication among referring agents, and establishment of youth corners in some hospitals in Zimbabwe to address concerns of privacy.

**Conclusion:**

The findings highlight that pathways to specialised mental healthcare in Ghana and Zimbabwe are inherently complex, hindered by intersecting structural, financial, and social barriers. To mitigate these challenges, a multi-faceted strategy is essential. This should include systemic interventions such as task-shifting, the implementation of streamlined referral tools, dedicated youth-friendly services, and concerted efforts to reduce stigma and costs, thereby fostering more equitable and timely access to care.

## Introduction

The prevalence of common mental disorders (CMDs) such as depression and anxiety among young people under the age of 24 is high, with at least 20% affected globally [1]. CMDs are the largest contributor to the burden of disability-adjusted life years (DALYs) for those under 24 in high-income countries and the seventh-highest contributor in low-and-middle- income countries [2]. CMDs are also linked to a range of problems among young people, including physical illness, risky behaviours, poor interpersonal relationships, and underachievement in both education and employment [3,4]. These negative effects can persist throughout the life course of a young person, posing serious consequences [5]. In sub-Saharan Africa (SSA), where 65% of the population is under 25, young people face a heightened risk of mental illnesses due to compounding factors like poverty, conflict, and HIV/AIDS among others [4]. Although limited, the available data suggests high prevalence of CMDs among young people in SSA, with an estimated 27% of young people aged between 10 and 19 and suffering from depression and 30% from anxiety disorders [6].

In Zimbabwe, at least 27% of young people aged 15–24 suffer from depression [7]. A recent study suggested that the true burden of depression in young people could be higher due to compounding factors like the COVID-19 pandemic, rising poverty, high unemployment rates and the economic crisis [8]. In Ghana, recent evidence shows the prevalence of depression among adolescents, estimated at 59.2% [9]. Suffice to mention that these cited studies did not include prevalence by sex. However, the reported high levels of depression underscore the importance of early identification and treatment of mental illnesses to prevent worsening of health conditions and subsequent undesirable social and treatment outcomes [10,11]. Several barriers impede young people's access to timely mental healthcare, including limited resources, lack of trained specialists, lack of culturally appropriate interventions, societal stigma [12,13] and complex help-seeking pathways [14–17].

Pathways to care are defined as the sequence of contacts or care points people use when seeking help [18,19], and are influenced by multiple intersecting individual, social, cultural, and systemic factors [19,20]. A pathway to care is therefore a dynamic process and can change over time and space because of influences from contextual determinants such as the prevailing economic environment at a given time [3,4]. The nature of mental health care and services available at each level of care within the pathway may also influence young people’s help-seeking behaviours [2].

In high-income settings, young people experience more direct routes to specialized care, while in low-resource settings, their pathways are often varied and complex, frequently involving faith healing and traditional remedies [2]. Such complex ‘pathways to care’ further delay access to specialised mental health care further exacerbating young people’s mental health issues [21]. Furthermore, research on young people's pathways to mental healthcare is scarce in low-resource settings such as Ghana and Zimbabwe. Establishing such pathways is essential to optimising access to timely diagnosis and care [22]. The limited availability of empirical evidence on young people's pathways to mental healthcare across SSA including Ghana and Zimbabwe is worrying considering the high prevalence of mental illnesses in this sub-population. The current study seeks to explore referral pathways for depression among young people living in Ghana and Zimbabwe.

## Materials and methods

### Study context and design

This study was conducted as part of formative work for the ‘African Youth in Mind’ (Y-MIND) consortium. This is a consortium of researchers from three countries of Ghana, Malawi, and Zimbabwe whose main aim is to adapt and test the feasibility of an intervention called the Y-MIND intervention [23]. The Y-MIND is a psychological intervention for youth depression and was tested and adapted in Ghana, in West Africa and Zimbabwe in Southern Africa [23]. For this exploratory descriptive qualitative sub-study, our goal was to explore the pathways that young people follow in seeking specialised or professional mental healthcare in the two countries. In Ghana, participants were young people aged between 15–18 years who were enrolled in the Ghanaian free senior high school system. On the other hand, in Zimbabwe, participants were in- school and out-of-school youths aged between 15 and 24 years. The difference in age groups between the two countries was necessitated by the distinct focus of each setting. For example, in Ghana, schools were purposively chosen as study sites, aligning with the school-going age range and the project’s efforts to strengthen existing school-based mental health systems. In contrast, in Zimbabwe, the study focused on clinics to explore how to adapt the Friendship Bench intervention model for youth depression.

### Study setting

The study was conducted in Navrongo (Ghana) and Harare (Zimbabwe). Navrongo, is a town in the Upper East Region of northern Ghana with a population of about 100,000, whose exact prevalence of CMD is not known. In Navrongo Ghana, qualitative data collection took place between January and May 2023 within the Navrongo Health and Demographic Surveillance Site (NHDSS) area. Harare, Zimbabwe’s capital city has a population of about 1.6 million people, and a relatively high prevalence (37.4%) of CMDs among young people aged between 13 and 24 [24]. Data collection in Harare took place between December 2022 and September 2023.

### Study team

In Ghana, the interviews were conducted by four trained and experienced qualitative research assistants (RAs) (one female and three males). The RAs were all multilingual in Kasem, Nankani, and English and had a minimum of bachelor’s degree in social sciences, with two to five years’ experience in conducting qualitative research. In Zimbabwe, data were collected by three RAs (1 female and 2 males) who were bilingual in both Shona and English and had a minimum of bachelor’s degree in social sciences. They all had vast prior qualitative research experience. Prior to data collection, all the RAs in both countries received initial training on qualitative research skills and on the study protocol, which included study aims and objectives, data management, participant recruitment, and research ethics.

### Participant recruitment

Purposive and snow-ball sampling techniques were used to recruit study participants in both countries, with invitations extended telephonically or via email using study participant information sheet and informed consent procedures, with assent obtained for those under 18 years. In Ghana, the Municipal and District Directors of Health and Education Services at the Ghana Education and Health ministries were purposively approached and in turn, they recommended other key informants through snowball sampling techniques. This led to the recruitment of five policy makers (3 in the Kasena-Nankana Municipality and 2 in the Kassena-Nankana West District). Similarly in Zimbabwe, the Director of Mental Health Services at the Ministry of Health and Director of School Psychological Services at the Ministry of Education were initially approached and asked to recommend other key informants, leading to the recruitment of five policymakers. In both countries, healthcare workers were recruited by approaching nurses in-charge at district hospitals, who recommended other informants, resulting in 22 recruited. School staff were recruited through recommendations from school heads, leading to 39 recruited in both countries. For lay health workers, their supervisors were approached who recommended other delivery agents, leading to 20 recruited in Zimbabwe at the Friendship Bench, and six in Ghana at healthcare centres. Finally, faith-based and traditional healers were recruited through mental health coordinators familiar with their work, resulting in six recruited. The information is summarised in Table 1 below.

**Table 1:** Socio-demographic profile of participants.

Category	Zimbabwe			Ghana			Total
	N	Gender		N	Sex		
		F	M		F	M	
Young people who received mental health services	15	7	8	12	8	4	27
Young people who did not receive mental health services	10	7	5	33	17	16	43
Healthcare Workers	4	4	0	18	10	8	22
Lay Health Workers	20	20	0	0	0	0	20
Policymakers	5	3	2	10	3	7	15
Faith-based healers	0	0	0	2	2	0	2
Traditional healers	0	0	0	4	1	3	4
School Staff	13	9	4	26	6	20	39
Total	67	48	19	105	47	58	172

### Inclusion and exclusion criteria

Inclusion criteria for the study included young people aged 15–18 in Ghana and 15–24 in Zimbabwe who were able to provide informed consent and assent for those under 18. The young people with lived experience had received formal care for symptoms of depression. The criteria also included individuals involved in the delivery or implementation of mental health services for young people in Ghana and Zimbabwe. These included professionals or volunteers such as healthcare workers, lay health workers, policymakers, school staff, traditional, and religious healers, all aged 18 and above and were willing to participate. Exclusion criteria included young people with an active mental health condition, determined by a previous clinical diagnosis of a severe mental health condition that could impair their ability to engage in the study. Moreover, in both countries, individuals involved in the delivery or implementation of mental health services for young people but had not lived in their community for at least 12 months were excluded.

### Data collection

In both countries, Key informant interviews (KII) were conducted with healthcare providers, policymakers, faith-based and traditional healers, lay health workers, and school staff. In-depth semi-structured interviews were utilised for young people. Additionally, in Ghana, separate four focus group discussions (FGDs) with students who had no history of mental illness were conducted. The interview guides included a core list of open-ended questions and anticipated follow-up questions, ensuring that the RAs asked all participants a minimum set of identical questions to collect credible and comparable data (included in the Supporting Information S2 to S9) For example, the topic guide for nurses covered key topics such as referral processes (e.g., “What happens when lay health workers or teachers refer to you a young person who needs a higher level of mental healthcare? Walk me through that process”). For young people, one of the central questions was around their experience with care: “How and why did you start going to the Friendship Bench/? How did you get referred here? (Draw out the process)?

In Ghana, interviews were conducted in English, *Nankani* and *Kasem*, and in Zimbabwe, interviews were conducted in English and *Shona*. Data collection continued until data saturation was reached. All interviews lasted for an average of 45–60 minutes and were conducted face to face. The interviews were audio-recorded and transcribed verbatim. Transcriptions were then translated from *Nankani/Kasem* (Ghana), and *Shona* (Zimbabwe) to English for analysis. Transcripts underwent quality checks including backtranslation to ensure that the meaning was maintained and were anonymised to ensure privacy and confidentiality of the participants.

### Inclusivity in global research

Additional information regarding the ethical, cultural, and scientific considerations specific to inclusivity in global research is included in the Supporting Information (S1 File).

### Data analysis

Verbatim transcription of audio data and translation to English (and back-translation) was done by different research assistants. A team of researchers within the Y-Mind Consortium checked the transcripts for quality and accuracy. An initial codebook was generated from the topic guides and first interview transcripts. Each transcript was assigned to at least two of the researchers, who read the respective transcript to familiarise themselves with the data then independently coded against the codebook deductively. Additionally, new codes emanated from inductive coding of the data. All the codes were thus grouped into themes. Suffice to mention that during the coding process, regular intercountry study team meetings were held (led by a social scientist, MK) to discuss progress in coding as well as the evolving codes and themes. New codes were merged at regular intervals using the NVIVO 14 [23,25] collaboration cloud function, to ensure all coders were able to use the same codebook. For this, specific objective being presented in this paper, there was no prior discussion of a coding tree by the authors and themes were derived from the dataset. A few selected participants were approached to provide feedback on the findings, and their input was incorporated into the results.

### Reporting

In reporting this study, the COREQ (COnsolidated criteria for REporting Qualitative research) checklist was adhered to, as a guiding framework [26]. Direct quotations of participants have been presented and identified with a participant’s code and country of origin.

### Trustworthiness

All the four pillars of trustworthiness, which include credibility, transferability, dependability and confirmability, were adhered to throughout data collection, analysis and writing of this manuscript. Thus, we believe our study findings have been accurately presented, and that they reflect the participants’ narratives. To that extent, each theme is supported with relevant participants’ quotes. To verify accuracy, we shared the findings with a few selected participants to confirm that we have reported what was said by them. To enhance transferability of our findings, we have provided contextual description of study participants and the study setting. Furthermore, our methodology is clearly defined and traceable. Additionally, the data analysis was done independently by PM and RM and a senior researcher (MK) audited the same.

### Ethical considerations

The study was approved by the Medical Research Council of Zimbabwe (Ref: MRCZ/A/2965), Navrongo Health Research Centre Institutional Review Board (Ethics Approval ID: NHRCIRB480), Ghana Health Service Ethics Review Committee (GHS-ERC Number: GHS-ERC:026/07/22) and the Kings College London Research Ethics (Ref: HR/DP-21/22–32917). To gain access to selected schools, approval was also sought from the Ministry of Primary and Secondary Education in Zimbabwe, and the Ghana Education Service (Ref: GES/DD-G/Q&A/24/016) in Ghana. Participation was voluntary, and written informed consent/assent was obtained from all participants. Those ≥ 18 years signed their own consent forms, while those < 18 years provided assent alongside parental or guardian consent. Anonymity and confidentiality were ensured by omitting identifying information from transcripts and securely storing data. A reimbursement allowance of US$10 was given to all participants to cover transport and related costs. For the sake of transparency in reporting this research study which was performed outside the primary (first) author’s country, as per requirement of this journal, we have attached the Inclusivity in global research checklist (Supporting Information S1). This has been done to ensure that our reporting adheres to high standards for research ethics and authorship.

## Findings

Three main themes emerged from this qualitative analysis to address the question: what are the referral pathways/trajectories for young people to access specialised care for depression in Zimbabwe and Ghana? These themes and subsequent sub-themes are presented in Table 2.

**Table 2:** Themes and sub-themes.

Theme	Sub-Theme
1. Supply factors – Availability and nature of specialised mental healthcare and services	a. Counselling services b. Other related services
2. Systems factors – Young people's trajectories for specialized mental health services	a. The role of multiple first points of contact b. The structural pathways within formal care c. Indications for referring young people d. Parental involvement in the referral process
3. Navigating the referral pathways – Referral barriers and enablers	a. Existing referral barriers b. Existing referral enablers

### Supply factors – Availability and nature of specialised mental healthcare and services

#### Counselling services.

Various types of mental health services such as counselling were said to be provided to young people with depression in both countries before a referral is initiated. Provision of some form of support before referral was made, especially when providers’ efforts were not yielding to improvement in the mental health condition of the young person as described by a nurse below:

*“The major issue is, if someone comes here and we realize they have a mental health problem, they are counselled. After being counselled we then try to address the problem, if it’s impossible they will then need a referral to the higher health institutions like Harare Hospital that have psychiatry units.”* (Nurse, Zimbabwe)

The frequently mentioned service provided to young people with depression in both countries was counselling, which is usually provided by lay health workers, community health workers (CHW), nurses, general teachers and guidance and counselling teachers in schools. Counselling was noted to be available to both in-school and out-of-school youths. For out-of-school young people in Zimbabwe, the most cited type of counselling provided was the friendship bench which is provided by CHW and lay health workers. *“Some of them we have counselling sessions with them, if it’s someone who is not violent, who has depression but is not violent we sit with them on the bench and have sessions with them”* (Lay health worker, Zimbabwe). In Ghana, the counselling services are provided by either nurses, general teachers or guidance and counselling teachers. *“I do my counselling and also refer to the guidance and counselling unit, and sometimes if the need be, we refer [them] to the psychiatric hospital for them to go for further counselling*.” (School Nurse, Ghana).

#### Other services.

Additional services that were provided before making referral included mental health screenings, school psychological services, school health programs like health pep-talks, and the monitoring of disorders for those with existing conditions and de-escalation services:

*“If a client comes, the client might get here in an acute phase, and they could be violent, right? So, with that patient what we do is that we calm down the patient then we write on the cards then we refer to a psychiatric unit”* (Nurse, Zimbabwe)

Through the school health pep-talks, which are aimed at creating awareness about mental health, students with mental health problems are identified and are either treated or referred for further management. “*For school, when you go for school health programme and you identify an issue, … the health teacher takes down the names of the affected students and then talk to them*” (Educational Technical Officer, Ghana). *“You know, from the school, when we go for the school health service like this, and we identify some cases we refer them to the health centres. So, if they get there and they think they cannot manage the case that is where we may need to contact the specialist*” (Lay Health Worker, Ghana). Health talks sometimes are provided by other key players like the Police. “*They [the Police] even address the children and even show them samples of how the drugs look like”* (Teacher, Zimbabwe). This helps the leaners to be aware and stay away from the same in case some peers give them to take. The services are provided by a multidisciplinary team to ensure that the care is holistic. Hence why key players like the police and other stakeholders like Non-Governmental Organizations (NGOs) take part. For instance, it was found that sometimes, young people are referred to NGOs for social welfare support. *“We have got other organisations like [name withheld] who come here-and-there to assist us. And the social welfare sometimes. Those are the ones that help us also when we handle those issues.”* (Education Policymaker, Zimbabwe)

Suffice to mention that the services outlined above are related and some of them happen concurrently. For instance, during health talks, the students are also provided with screening services. “*We have also done trainings with MSF, Medecins Sans Frontieres to train teachers. So this is meant to just be able to screen, to screen their students for mental health and then refer thereafter” (Health Policymaker, Zimbabwe)*

### Systems factors – Young people's trajectories for specialized mental health services

#### The role of multiple first points of contact.

When analysing the pathway and the trajectory taken by the young people, the data illustrated the presence of multiple first point of contacts (FPOCs) that are utilized by young people from both countries, before they get specialised mental healthcare. These FPOCs include both formal agents (biomedical providers) and informal agents (faith-based healers and traditional healers).

Suffice to mention that before getting either into the formal or informal routes for proper care, for young people, the very first point of contact is usually their peers. Its either the young person with the mental health issue will approach a peer or the peer will notice some changes in their peer and approach them. However, there is an extent to which they disclose their issues to a peer because they are concerned with their privacy. According to some participating young people, even though they think their peers are better placed to understand them, they don’t have the skills to maintain confidentiality. “*What I am saying is I can’t confide in him or her [a peer] 100% but l think they understand better because they are my peers. They know the things I am just explaining …and they understand better. … but l confide better with a therapist” (YP 3, Zimbabwe).*

Faith-based healing is related to religion where prayers, worship, prophetic revelations and pastoral/imam counselling are utilised to provide some therapeutic elements to a patient. On the other hand, traditional healing (TH) involves spiritualism, concoctions, and cleansing rituals. TH is utilised not only to provide treatment but also to find the root cause of the suffering. Study participants highlighted that young people's choices on which FPOC to approach depends on both the context in which the mental health condition has started and the circumstances surrounding the condition. *“If it’s from the community, we have health promoters. They are the ones that refer people [To clinics]. In schools… nurses would go, and they would look for those with mental illness then they refer them to us.”* (Nurse, Zimbabwe).

*Most key informants indicated that* the most preferred FPOC for all, including young people, was the formal care followed by informal care. Worth noting is that in Ghana, even the informal care providers support the notion that clients should first utilise the formal care before approaching them. *“I think they should first be taken to the health professionals”* (Faith-based Healer, Ghana). Even after the case has come to their attention, some informal players still refer to the formal sector where necessary. *“You know the [traditional] doctors are of different types, if the person is here [with us] and the condition is not better, it is good to send the person forward [to formal care]”* (Traditional Healer, Ghana).

It was found that the faith-based entry points into the mental health treatment pathway are often utilized when the patients observe no visible improvements in their condition while utilising the formal/biomedical approaches. As such, the informal care providers leave their door open in case of such scenarios. *“If they realise that they will not be able to handle [the case] then they can let we, the faith-based healers to also come in, to support, to see if they can be treated.”* (Faith-based Healer, Ghana). Contrary to this common scenario, a young girl narrated her experience when she was initially taken to a faith-based healer and then proceeded to formal care when they did not notice any improvement to her suffering. “S*he [her mother] told me I cannot go to school because we are to go to some place then we will now go to the school …. she sent me to a local church… they did somethings over there, … they prayed and …when we went home it happened that I wasn’t able to sleep in the night then we got up again and we went to the hospital*” *(FGD participant 1, Female YP, Ghana)*.

While most of the participants perceived that the formal care is more preferred and utilized, some thought that the informal care was more favoured because informal care providers take time in explaining the root cause (aetiology) of the problem and that the waiting time is minimal compared to the formal care. It is not clear however, if people use both the formal and informal care simultaneously. The Fig 1 below illustrates the trajectories that the youth in both countries take in pursuit for specialized or formal mental health care. The figure shows that young people move from the community into the mental health care system using two main routes of formal (a) and informal care (b). Once they are in the mental healthcare system, some move from formal to informal (d) and from informal care to formal care (c).

**Fig 1:**
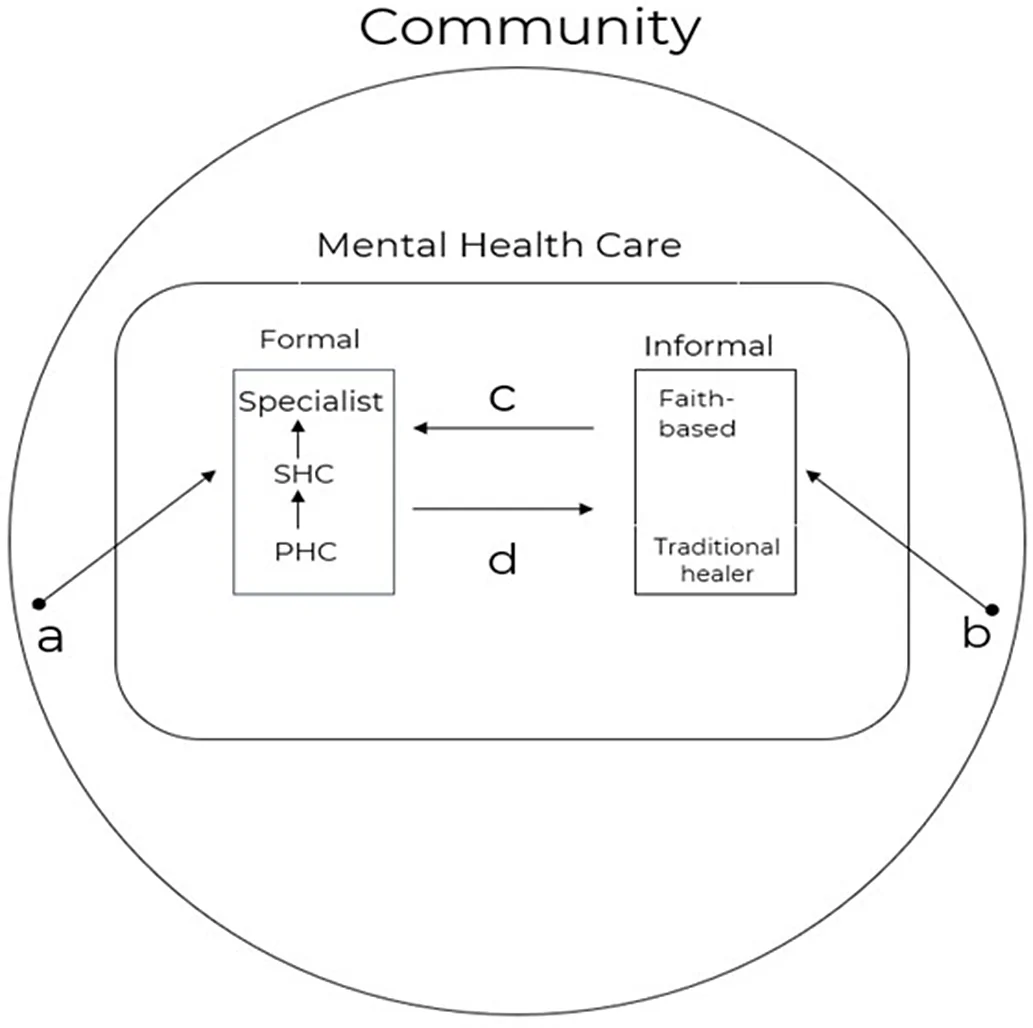
Pathways to mental healthcare taken by young people in Ghana and Zimbabwe.

#### The structural pathways within formal care.

Notably, in both countries, the formal care system is structured into primary health care (PHC), secondary health care (SHC) and tertiary/specialist care where clients, young and old, move in a hierarchical or pyramidical fashion.

In both Zimbabwe and Ghana, we noted that for school-going adolescents and young people to get specialized mental healthcare they must start from the school, then from there they get referred. For instance, in Zimbabwe, the young person has to go through some levels of care before meeting a specialist: *“So we have the referral pyramid, which is stratified from primary healthcare, then going secondary. Quaternary and quinary. So, we have gone further in developing a service directory so that schools can actually refer people*” (Health Policy maker, Zimbabwe). Similarly, in Ghana the school-going young people are referred to primary health care which then refers them to a Health Centre (HC) and from the HC they are referred to a hospital to be seen by a GP, as a gatekeeper and then a specialist, if need be. *“For our CHPS [Community-based Health and Planning Services], let’s say the primary health care facilities they normally refer to the health center then the health centers too would then refer to the hospital. Then from the hospital they would then arrange the specialist. Also, for our system, we are not supposed to just refer directly.”* (Lay Health worker, Ghana).

Within the school system, before a student is referred, data showed that young people usually go through a long, tedious and convoluted process before referral is made for care at a referral health facility. A school nurse described the need to follow protocol established within the school establishment before referring a student. *“In school, for instance, when there is a mental health issue or when I have something or a challenge, I try seeing the health master and then I discuss the problem with him. If he is not there or he can even be there and say ‘ok, let’s go and see the senior housemaster’ and from there we see the Assistant head of academics and then from there if it will get to the headmaster before, we move to the health facility. That’s how it is”* (School Nurse, Ghana). The same was also noted to be the case in Zimbabwe: “*At this school we just take you [the YP] straight up to the senior woman or senior master. But if it’s too much for the senior master and woman, I just take it straight to the headmaster and inform him of the issue. After that, he will see what he can do or even call the parents if there is the need to” (Teacher 03, Zimbabwe).* Fig 2 is an illustration of the trajectory that is taken by school-going adolescents in seeking specialistic mental health care in both countries. The shape of the ladder is in a form of a pyramid representing the number of youths who go up the ladder. Many get assisted within the school system, and very few proceed to the specialist. However, first level consists of multiple steps, representing the complex processes that young people go through before being referred to the next level, i.e., the Primary Health Care. Study participants collectively acknowledged that this long pathway contributes to delay in seeking healthcare and consequently loss to care within the referral cascade as the youth are likely to get frustrated due to long waiting hours.

**Fig 2:**
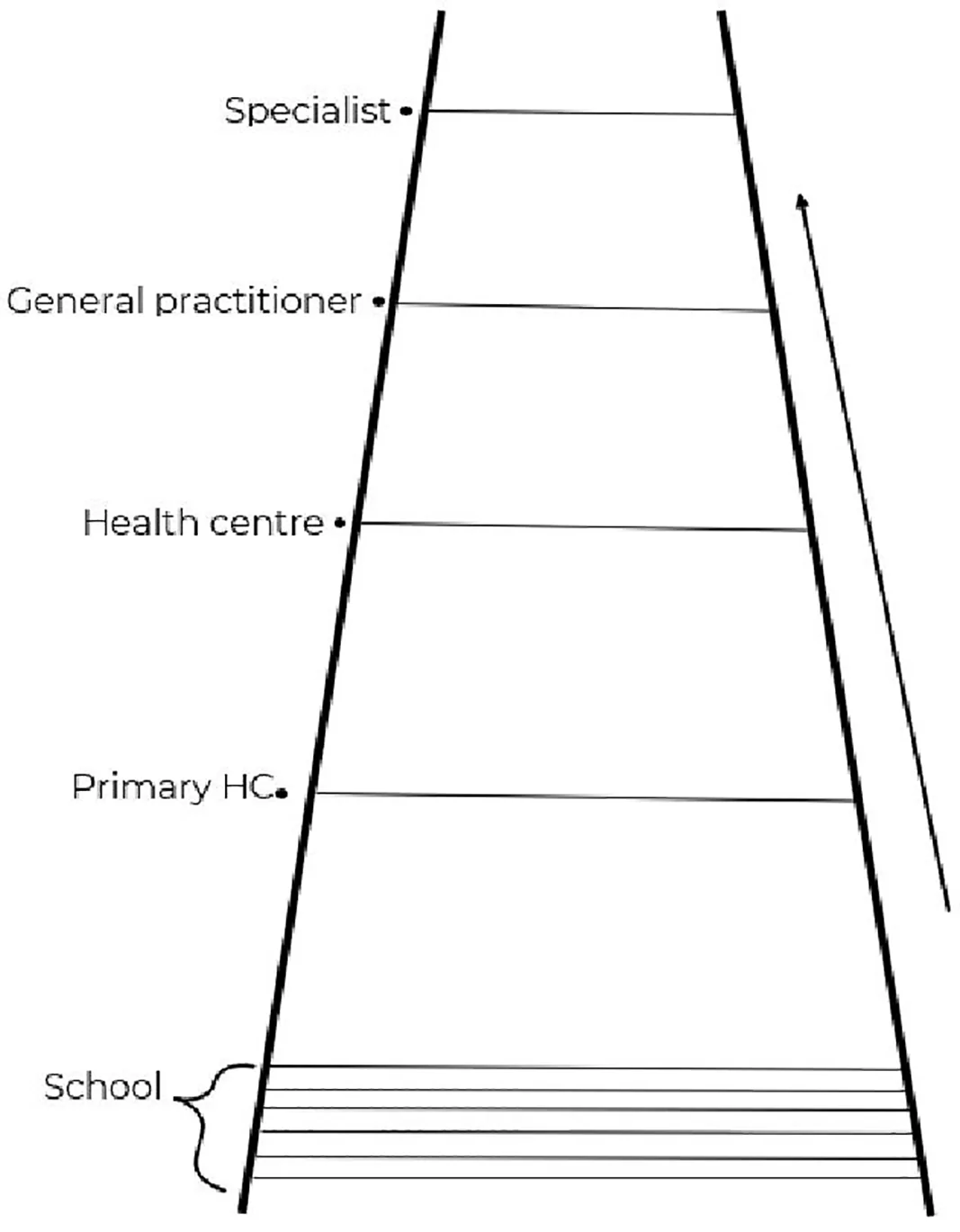
Trajectories for School-going adolescents in Ghana and Zimbabwe.

#### Indications for referral.

There are various reasons that the youth are referred from one level to the next within the referral pathway. These reasons include but are not limited to the severity and complexity of the case, especially if psychotic symptoms are present. “*There are some cases where they are hallucinating, so sometimes, we can ask if they have been to the general practitioner if not please try or we might have contacts of those we know are psychologists we can always refer to them*.” (Lay counsellor 06, Zimbabwe); the type and nature of comorbidity; the type of care available at that level among others. A young person may also get referred when their condition poses a risk to others and de-escalation is required. *“For those with severe depression for example those who are violent, we can’t sit with them on the bench, we refer them to the nurse then they get help there”* (Lay Health Worker, Zimbabwe).

Within the formal treatment pathway, young people with complex issues get referred to other external players for holistic care. *“…if there is a need to inform the police, then we do likewise. Normally at this school, most of the time we work with the police especially on the issues related to drug abuse.* Our data further indicated that young people usually do not have a voice in decision-making regarding their condition especially where to be referred. Disease-severity, type of comorbidity (for example cases involving drug and substance abuse) and perception of the effectiveness of a pathway or a service influenced the decision of teachers and /or parents in choosing a pathway for a young person.

#### Parental Involvement in the referral process.

It was observed that parents and guardians play a crucial role in the referral pathway of their children, even though this is not always the case. *“When it gets to where you need to refer, then you have to involve the parents, [or] guardians, let them understand that, this is why we are referring ‘so you need to take him or her to the specialist early for treatment’...”* (Educational Technical Officer, Ghana). However, parents are not forced, they are simply enlightened but the decision of where to take their child to, lies in their hands. “*For traditional/ spiritual healers, it is the parents not school authorities who refer the young people [there] (Teacher, Zimbabwe).* It was found that in both countries, policies and/or guidelines exist on how and where teachers can refer a young person. “*So, the guidelines are there if it’s any other form of depression or anxiety that one you can try to solve within the school but if it’s something that comes from teenage pregnancy or drug abuse, the parents must be involved” (Teacher Ghana).* The same was also found in Zimbabwe: *“For example, some people refer you to a prophet who helps but it’s not allowed [by the school]. Otherwise, the parent is the one who might go to the prophet, who go to the traditional healer, it’s not the school, it’s not”.* (Teacher, Zimbabwe)

Regarding the timing of the involvement, it was noted that parents are mostly involved in severe cases when critical decisions must be made. *“When it comes to pregnancy, where there’s anxiety and depression, when we talk of drug abuse, with those ones, we equally invite the parents to come immediately.”* (School Health Teacher, Ghana). Sometimes the parents are not necessarily required to present themselves at the school. If anything, they are just informed for them to be aware of the situation. *“If there is a need for the parents to come, we will now let them come, mostly this is what we do.”* (School Nurse, Ghana).

However, parental involvement works where communication is not a problem. For example, some young people, especially those far from their parents, complained that where their parents are to be involved, it becomes difficult due to communication gaps. “*It is not all of us that our parents have phones, me for instance my mum doesn’t have a phone. I have to call my uncle house so that they will inform my parents” (FGD participant 7, Female YP, Ghana)*.

Parental involvement also depends on the type of agents involved. For example, when the case involves the police, parental involvement is a must. Among other things, the role of guardians and parents is to decide where they want their child to be referred to. *“...but sometimes they might even call the parents depending on how the condition is, they might call the parents to come and take the child and when they get to the hospital it is now out of the school.”* (Special Education Coordinator, Ghana)

### Navigating the referral pathways – Referral barriers and enablers

#### Existing referral barriers.

Young people faced numerous challenges in relation to the referral process. The most common challenge mentioned by the study participants mainly those from Ghana, was finances. This was primarily related to the cost of transporting a young person to a facility and the cost of medical services, i.e., insurance. *“The commonest one is the transportation or means of transport. If the school pick-up goes out then I have to rely on the motorbike and then sometimes you will refer the student and then you realised that student’s health insurance is not working”* (School Nurse, Ghana)*.* In a situation where there is no transport for the students and no medical insurance to cover for the students’ medical bills, teachers shoulder the burden, and they are forced at times to pay from their personal savings. *“…like I would take the [young] person there but wouldn’t stand there for them to bill me because I don’t have money in my pocket. So, everybody is afraid. When you take the person there, the cost that is coming, you are going to bear it, the students don’t have anything. So, it is a challenge”* (Guidance and Counselling Teacher, Ghana). This was echoed by a YP: “*Even during the day, when you ask the teachers to help, they will say their fuel and in case they say you should pay something, they can’t use their salary for that, so you have to call home…The teachers are always afraid that when they send you to the clinic, they may have to take care of your bills”* (FGD participant 7, Female YP, Ghana).

Apart from transportation challenges, a nurse in Ghana mentioned that in their whole region there is just one specialist psychiatrist to be referred to. This usually leads to long waiting time and this contributes to loss to follow-ups within the care cascade. Lack of specialised youth-friendly services was also described as a barrier. *“What they don’t like, they want their own space but if it is for them to come and queue, the aunt might be there, the uncle’s wife or somebody is there, they will not come.”* (Public Health Nurse, Ghana). Apart from being concerned with their privacy, as youths, sometimes their autonomy is forfeited. It was noted that, even where more options are available, authorities just direct the youth to a particular service provider without discussing alternatives with them and this poses a challenge for them to abscond from care. *“So, if you say, ‘go to the counsellor’ he will never agree to it so the option should be given to the student to know ‘where I want to go for the services’”* (Mental Health Nurse, Ghana)*.* Negative attitude by some key referral agents was also identified as a challenge. *“So, I think referring from primary health system to sometimes the regional hospital we have office challenges, but mostly it is the attitude of the staff, some of them the response is very good, but some too is not good.”* (Public Health Nurse, Ghana)*.* Unfortunately, some young people also have negative attitudes against mental healthcare. The negative attitude by young people is perpetuated by stigma. In some parts of Zimbabwe for instance, people think attending therapy is for those who are ‘crazy’. As a result, when a young person has been referred to a therapist, they immediately develop some resistance. *“If you tell them to go see a therapist they might start questioning if they are going crazy because that’s what they believe seeing a therapist is for.”* (Friendship Bench Counsellor, Zimbabwe). Similarly, in Ghana, whenever, a young person is referred to a counsellor, people misjudge the young person. *“When you are going to see the counsellor, people will be talking about you, they will be saying bad things about you, they will think that you have done something very bad, even when you have finished talking to the counsellor, they will now be pointing hands at you. They will not even associate themselves with you, because they will see you to be a bad person”* (FGD participant 3, Female YP, Ghana)*.*

Lack of a conducive environment and infrastructure for the counselling to be done was also pointed out by the participating young people as a barrier to attending counselling when it has been indicated*. “There are no offices there for them to be sitting there to be counselling people, even we the students we do not have place to sleep let alone an office for them”* (Female FGD participant 3, Ghana).

#### Existing referral enablers.

Despite the referral barriers described earlier, several referral enablers were identified. These referral enablers include the availability of a pre-referral note or booklet which is widely used to expedite the referral process in Ghana. Due to lack of access to existing medical insurance schemes by some young people, some NGOs provided free medical services for such youths. “*There was some money that I had to pay before they discharged us [from the hospital] so the [mentioned a name of an NGO] people came and supported me to do that and I went home”* (Young person, Ghana). Some health facilities in Zimbabwe responded to the challenge of lack of youth friendly services by providing youth corners where youth-friendly services are provided. *“For young people there is an adolescent corner and there would be adolescent nurse to monitor them. So, we came up with a way that ‘adolescents [who] don’t want to mix with other groups, especially the elderly, they feel uneasy, they won’t disclose what they would’ve come for’. So, if they get treated in their own group they march in and meet up with the nurse, who then treats them and if they have problems that needs referral they get referred. If they want to be helped at local level, they then get help and that is really working out*.” (Nurse, Zimbabwe). A similar arrangement also exists in Ghana. “It is called adolescent health [corner] so we always go there and have our free chat with the nurses over there and then I go to school” (YP, Ghana).

Good communication and interaction among referral agents facilitate the referral process, thus minimizing delays for young people. In Zimbabwe for instance, a service directory was developed to ensure that referring officers know where to refer a young person. *“So, we have gone further in developing a service directory so that schools can actually refer people.”* (Health Policymaker, Zimbabwe). In Ghana, when a student has been referred to a facility, the referring officer alerts the receiving end so that they are aware of the student's coming so that the student does not need to queue. *“We have this referral book and the register at the facilities, so when we are referring a case, we call the receiving facility telling them about the case, when the case gets there, the person would not need to join a queue but will just direct the person to where they would get the attention”* (Lay Health Worker, Ghana).

Another enabler relates to the barrier of lost to follow-up. In Ghana, it was observed that unlike before, referring institutions follow-up with referred cases to ensure that the young person indeed complied with the referral. *“The staff is still doing their best. They will counsel you and follow up the next day to check on you to see whether you have gone or not”* (Educational Technical Officer, Ghana). In some cases, community leaders and influential people in society are utilised to motivate young people and their guardians to adhere to the referral. *“When the officer goes to the house two times and sees that they are not making an advance to go [where they have been referred to], we involve the opinion leaders or the assembly member maybe when they also talk to them, …so when you involve those people maybe the assembly member or chief of that community, it helps, some do listen to them”* (Educational Technical Officer, Ghana).

## Discussion

This study looked at the trajectories or pathways that young people with depression from Ghana and Zimbabwe take when acquiring specialised psychiatric services. We found similar results in the two countries. For example, it was found that for young people to access mental health services, they approach various first point of contacts and that multiple agents are utilised, leading to a long pathway and delayed care, sometimes. A retrospective study of patients of all age groups seeking mental health services at a public psychiatric facility in Ghana found that the commonest first point of contact for about half (48%) of the patients was the informal care (either faith-based or traditional healers or general practitioners) [27]. This means that unlike the 52% who had a direct pathway to formal care, 48% of the participants had an indirect or complex pathway to formal care. These findings [27] agree with what other studies found on the pathway to mental healthcare in Ghana [2]. For example, a systematic review on the pathways of mental health treatment in Ghana indicates that faith-based and traditional healing pathways, unlike the biomedical pathway (BMP), are commonly used as FPOC [28]. In Zimbabwe, the situation is not different from the one found in Ghana. However, a study done in Harare, Zimbabwe more than two decades ago by Patel V et al (1997), on the pathways to primary mental health care found that most patients utilise more than two pathways of care simultaneously; mainly BMP and traditional healers (TH) [29]. It was noted that initially, patients approach the biomedical care pathway. When this care did not meet their expectations, patients resort to using another BMP and traditional healers simultaneously or traditional healers alone [29]. Another study done twenty-seven years later in the same setting (Zimbabwe) found similar results [30]. More recently, Mlambo Nefasi (2024) did a qualitative study to explore the pathways to care in the Zimbabwean mental health sector and found that multiple pathways exist and that while some clients simultaneously use two pathways, the majority use three pathways depending on the timing of the day [30]. For example, the formal/BMP is utilised in daylight while the other pathways especially, faith-based healers (FH) and TH are mostly utilised covertly during the evenings after the BMP has been consulted during the daytime [30]. Suffice to mention that these referenced studies were for all age groups and not specific for young people, as studies exclusively on the pathways for young people are scarce. Thus, based on our study findings, young people’s FPOC are similar to those of adults in both Ghana and Zimbabwe. The only difference however, was that while multiple FPOCs are utilised simultaneously in Zimbabwe, in Ghana, people use a single FPOC and move to another if they do not observe or experience any change.

In the two studies above, both Patel et al (1997) and Mlambo Nefasi (2024) [29,30] considered the factors that contribute to the choice of a particular pathway, and it was observed that sociodemographic and economic status and the cost of a particular pathway, health status of an individual, perception of the effectiveness of a particular pathway and severity of the illness are some of the contributing factors [29,30]. These findings are similar with our study findings where disease-severity as well as perception of the effectiveness of a pathway or services received influenced the decision of parents in choosing a pathway for their child. Additionally, Patel et al (1997) further, found that most people in Zimbabwe prefer traditional healers to the biomedical care because the former at least provided them with an explanation of the aetiology of the disorder [29]. This is related to our finding where a teacher from Ghana was of the view that faith healers are more preferred because they spend more time navigating the probable etiological factors with their clients. Even though this may not have a strong evidence-base, it is plausible that spending more time to explain the probable cause of a patient’s condition is likely to increase the time that a client spends with a service provider, and this has a potential of improving their outcome [31]. Moreso, spending more time with a client entails that a client is given ample time to ask question which may help them clear their minds. Common misconceptions and beliefs about the aetiology and prognosis of psychiatric disorders have also been identified as factors that determine the choice of a pathway [2]. Some of the reasons given are that traditional and faith-based healers engage well with their clients and that due to shared spiritual and cultural belief with the clients, it helps them to easily build rapport and trust. Furthermore, these studies found that traditional and faith-based healers are relatively cheaper, affordable and accessible [29,30,32]

Our study found that the gatekeeper referral system is commonly utilised both in Zimbabwe and Ghana. In this system, cases that require specialists’ intervention come mostly from general practitioners (GPs). The GP are gatekeepers who receive referrals from all the referral agents including teachers, community health workers, the police and other governmental and non-governmental organizations. However, this is different from what is practiced elsewhere. For instance, a study on the referral patterns to outpatient child and adolescent mental health services (CAMHS) from Denmark, found that there are two main primary referral sources for specialists- GPs who are the main referral source for general psychiatric conditions and educational psychologists who are the primary referral source for developmental disorders [33]. The disadvantage of the gatekeeper system is that it contributes to delayed treatment [22] which has implications on health-seeking behaviours and continuity of care. Thus, increasing the already existing wider treatment gap in low- and middle-income countries [34]. As a remedy to reduce this wide treatment gap, the World Health Organization (WHO) introduced the task-shifting model where specialists delegate some of their roles to low-cadres healthcare workers like GPs and CHWs upon training them [35]. This is why, in both Ghana and Zimbabwe, as found by our study, lay health workers and GPs are being trained to provide specialistic services, under the supervision of the specialists- and this came as an enabler in our study findings. As illustrated in Fig 2, only few complicated cases are sent to the specialists.

This study further found that parents are minimally involved in the referral system. They are only involved when the cases are severe or when some external agents like the police are involved. A systematic review and meta-analysis of the impact of parental involvement in adolescent psychological intervention found that involving parents in the treatment of young people’s psychopathology improves the outcomes and its impact is greater than when parents are not involved [36]. In our study, some of the challenges that were found include lack of transport and medical insurance to cover for medical bills. We believe that involving parents would also help in addressing these two challenges. However, involving parents has its disadvantages. In our study, it was found that, parental involvement sometimes interferes with young people’s autonomy and privacy. As such, there is need to balance between parental involvement and young people’s autonomy and privacy. We are of the view that young people should be engaged in determining the extent to which their parents should be involved in their care to maintain their trust and engagement with mental health services.

While navigating the referral pathways, young people face numerous barriers that prevent them from accessing specialised mental healthcare. In Ghana, the following barriers were identified; direct and indirect costs of healthcare like lack of health medical scheme and transport to meet a specialist, inadequate mental health specialists, and negative attitude of some referral agents within the pathway. In Zimbabwe, only three barriers were identified which are related to the ones found in Ghana and these were stigma by both healthcare workers and the community at large, inadequate mental health specialists and lack of mental health youth-friendly services. A scoping review on barriers that impede access to mental health services for children and adolescents in Africa which included studies from six countries of Ethiopia, Mali, Egypt, South Africa, Nigeria, and Tunisia also identified some of the barriers that our study found like financial difficulties, stigma and negative attitude (expectations by the young people and their guardians that care services will be unavailable where they have been referred to) [37]. Additionally, as discussed above on the preferred FPOC, the scoping review also listed preference for traditional /alternative and complementary treatments in Africa as a barrier to seeking specialised and professional mental healthcare [37]. Other identified barriers not explicitly mentioned as barriers by our study participants but were mentioned by the scoping review include poor diagnostic skills by low-cadre healthcare workers thus delaying referral, refusal to seek help or get referred, parental denial and delay to consent their child to get referred [37]. It must be emphasized however, that the identified barriers above are not just unique to Africa. It has been found that barriers like lack of insurance coverage, stigma, and shortage of mental health specialists have also been identified in high income countries like the United States of America [38].

Our study further found that there are good communication and networking among the key referral agents. However, the cordial relationship was more prominent among formal key players like biomedical professionals and non-biomedical professionals like the police, social welfare, teachers, community health workers and some NGOs. In both Ghana and Zimbabwe, the relationship between the biomedical/formal healthcare providers and the informal providers like traditional- or faith- healers did not come out clearly from our data. When analysing a care pathway, one of the key things to be considered according to some schools of thoughts is that services and service providers must be well connected and coordinated within the system of care [39]. Key questions though, to consider are: 1. to what extent should these players be connected? 2. How should the biomedical professionals collaborate with traditional and faith healers for mental healthcare? A further study is therefore warranted to answer these questions as neither our study nor our literature review, were able to answer these questions. One of the major reasons why there is a problem of collaboration between biomedical professionals and traditional and faith healers is the existence of barriers to their collaboration. Some of the barriers that have been identified by studies in Africa and Asia include lack of trust between biomedical professionals and faith healers, power differential, disagreements over conceptualisation of disorders and recovery, methodological approaches in assessments and management and lack of resources as well as organizational support [32]. To overcome these barriers and promote effective and sustainable collaborations between the formal and informal care, there is a need for ‘bottom-up’ approaches using dialogue, community engagements and mutual learning between the two players [32].

The good communication mentioned above as an enabler to referals was identified in both countries. Specific enablers identified in Ghana were availability of financial support to the needy by some NGOs and use of influential leaders to persuade young people and parents who refuse to go where they have been referred. In Zimbabwe, country specific enablers/facilitators of seeking specialised mental healthcare included availability of youth-friendly service to cater for privacy concerns by the youth and availability of a service directory where a referring agent can find a nearby institution to refer a young person.

In summary, our study demonstrates that young people in Ghana and Zimbabwe navigate remarkably similar pathways to specialist mental healthcare, shaped by the same constellation of FPOC, barriers, gatekeeping structures, and enablers.

### Study implications

The findings from our study highlight that pathways to specialised mental healthcare in Ghana and Zimbabwe, as in most low-resource settings, are inherently complex, hindered by intersecting structural, financial, and social barriers. The implications of these study findings for practice are that for practitioners to increase mental health service-utilisation among young people in low-resource settings, there is need for them to consider and address these barriers. To mitigate these barriers, a multi-faceted strategy is proposed. This strategy includes but not limited to systemic interventions such as task-shifting, the implementation of streamlined referral tools, dedicated youth-friendly services, and concerted efforts to reduce stigma and costs, thereby fostering more equitable and timely access to care.

In terms of research, implementation strategies like stigma reduction or task-shifting could be tested and see if they can improve uptake of mental health services. Additionally, there is need to research on how and to what extent, can the formal and informal support systems can collaborate to promote mental health service-utilization among young people in low-resource settings.

### Limitations

The major limitation of this study is the variations in the age ranges between the two countries makes it difficult to make a logical comparison even though our main objective was not to compare the two study settings. However, this was necessitated because as mentioned, in Ghana, the study setting was in school as such, the students were in a different age range to those sampled in Zimbabwe. We believe that given our research question, the age differences could not have a major effect on our study findings.

## Conclusions

In conclusion, this study has highlighted the pathways to care, challenges faced and opportunities available to the youth when manoeuvring around the pathway as well as the services available to them within the pathway and to what extent their parents and guardians are involved in the referral system. These results are likely to inform policy by among other things, ensuring that task-shifting services are scaled up in the two countries so that mental health services are closer and accessible to the youth and their guardians within their communities. Furthermore, our study provides insights for further research on evidence of holistic pathways of multidisciplinary services in SSA, whose evidence has been limited across the globe as observed by a Cochrane systematic review and meta-analysis on the effects of clinical pathways on professional practice, patient outcomes, length of stay and hospital costs [40].

## Supporting information

S1 File Inclusivity in global research- checklist. (DOCX)

S2 File Adolescents who have used the youth friendship bench- Interview guide. (DOCX)

S3 File Village health workers – Interview guide. (DOCX)

S4 File High school students – Interview guide. (DOCX)

S5 File Friendship bench counsellors – Interview guide. (DOCX)

S6 File School teacher – Interview guide. (DOCX)

S7 File Ministry of health/Ghana health service officials – Interview guide. (DOCX)

S8 File Adolescents with mental health history – Interview guide. (DOCX)

S9 File Mental Health care service providers – Interview guide. (DOCX)

## References

[1] Mubeen Z, Fatmi Z, Hameed W, Asim M. Barriers and facilitators to accessing adolescents’ mental health services in Karachi: users and providers perspectives. BMC Health Serv Res. 2024;24(1):157. doi: 10.1186/s12913-024-10593-0 38302915 PMC10832177

[2] MacDonald K, Fainman-Adelman N, Anderson KK, Iyer SN. Pathways to mental health services for young people: a systematic review. Soc Psychiatry Psychiatr Epidemiol. 2018;53(10):1005–38. doi: 10.1007/s00127-018-1578-y 30136192 PMC6182505

[3] Patel V, Flisher AJ, Nikapota A, Malhotra S. Promoting child and adolescent mental health in low and middle income countries. J Child Psychol Psychiatry. 2008;49(3):313–34. doi: 10.1111/j.1469-7610.2007.01824.x 18093112

[4] World Health Organization. Guidelines on mental health promotive and preventive interventions for adolescents. 2020 [cited 2024 February 24]. Available from: http://apps.who.int/iris 33301276

[5] Seekles M, Twagira F, Alam A, Obasi A. Promotive and preventive interventions for adolescent mental health in Sub-Saharan Africa: a combined scoping and systematic review. BMJ Public Health. 2023;1(1):e000037. doi: 10.1136/bmjph-2023-000037 40017854 PMC11812691

[6] Jörns-Presentati A, Napp A-K, Dessauvagie AS, Stein DJ, Jonker D, Breet E, et al. The prevalence of mental health problems in sub-Saharan adolescents: A systematic review. PLoS One. 2021;16(5):e0251689. doi: 10.1371/journal.pone.0251689 33989357 PMC8121357

[7] UNICEF. The state of the world’s children 2021. 2021 [cited 2024 Dec 31]. Available from: https://www.unicef.org/reports/state-worlds-children-2021

[8] Ministry of Health and Child Care, UNICEF. A case for mental health of children and adolescents in Zimbabwe. 2023 [cited 2024 December 31]. Available from: https://www.unicef.org/zimbabwe/media/9016/file/A%20case%20for%20mental%20health%20of%20children%20and%20adolescents%20in%20Zimbabwe.pdf

[9] Kumbet SJ, Oseni TIA, Mensah-Bonsu M, Damagum FM, Opare-Lokko EBA, Namisango E, et al. Common adolescent mental health disorders seen in Family Medicine Clinics in Ghana and Nigeria. PLoS One. 2023;18(11):e0285911. doi: 10.1371/journal.pone.0285911 37971998 PMC10653403

[10] Javed A, Lee C, Zakaria H, Buenaventura RD, Cetkovich-Bakmas M, Duailibi K, et al. Reducing the stigma of mental health disorders with a focus on low- and middle-income countries. Asian J Psychiatr. 2021;58:102601. doi: 10.1016/j.ajp.2021.102601 33611083

[11] Schleider JL, Weisz JR. Reducing risk for anxiety and depression in adolescents: Effects of a single-session intervention teaching that personality can change. Behav Res Ther. 2016;87:170–81. doi: 10.1016/j.brat.2016.09.011 27697671 PMC5127737

[12] Wasil AR, Osborn TL, Venturo-Conerly KE, Wasanga C, Weisz JR. Conducting global mental health research: lessons learned from Kenya. Glob Ment Health (Camb). 2021;8:e8. doi: 10.1017/gmh.2021.7 34026238 PMC8127636

[13] Osborn TL, Venturo-Conerly KE, Arango G S, Roe E, Rodriguez M, Alemu RG, et al. Effect of Shamiri Layperson-Provided Intervention vs Study Skills Control Intervention for Depression and Anxiety Symptoms in Adolescents in Kenya: A Randomized Clinical Trial. JAMA Psychiatry. 2021;78(8):829–37. doi: 10.1001/jamapsychiatry.2021.1129 34106239 PMC8190694

[14] Ochuku B, Osborn TL, Nerima D, van der Markt A, Rusch T, Omune H, et al. Testing pathways to scale: study protocol for a three-arm randomized controlled trial of a centralized and a decentralized (“Train the Trainers”) dissemination of a mental health program for Kenyan adolescents. Trials. 2023;24(1):526. doi: 10.1186/s13063-023-07539-y 37574545 PMC10424401

[15] Abdulmalik JO, Sale S. Pathways to Psychiatric Care for Children and Adolescents at a Tertiary Facility in Northern Nigeria. J Public Health Afr. 2012;3(1):e4. doi: 10.4081/jphia.2012.e4 28299078 PMC5345448

[16] Bakare MO. Pathway to care: first points of contact and sources of referral among children and adolescent patients seen at neuropsychiatric hospital in South-Eastern Nigeria. Niger J Med. 2013;22(1):52–6. 23441521

[17] Kamau JW, Omigbodun OO, Bella-Awusah T, Adedokun B. Who seeks child and adolescent mental health care in Kenya? A descriptive clinic profile at a tertiary referral facility. Child Adolesc Psychiatry Ment Health. 2017;11:14. doi: 10.1186/s13034-017-0151-x 28293286 PMC5348803

[18] Gater R, de Almeida e Sousa B, Barrientos G, Caraveo J, Chandrashekar CR, Dhadphale M, et al. The pathways to psychiatric care: a cross-cultural study. Psychol Med. 1991;21(3):761–74. doi: 10.1017/s003329170002239x 1946864

[19] Rogler LH, Cortes DE. Help-seeking pathways: a unifying concept in mental healthcare. Am J Psychiatry. 1993;150(4):554–61. 10.1176/ajp.150.4.554 8465869

[20] Anderson KK, Fuhrer R, Malla AK. The pathways to mental health care of first-episode psychosis patients: a systematic review. Psychol Med. 2010;40(10):1585–97. doi: 10.1017/S0033291710000371 20236571

[21] Dell’osso B, Altamura AC. Duration of untreated psychosis and duration of untreated illness: new vistas. CNS Spectr. 2010;15(4):238–46. doi: 10.1017/s1092852900000079 20414173

[22] Kauye F, Udedi M, Mafuta C. Pathway to care for psychiatric patients in a developing country: Malawi. Int J Soc Psychiatry. 2015;61(2):121–8. doi: 10.1177/0020764014537235 24903683

[23] Jopling R, Attah D, Abas M, Adde KS, Goldsmith K, Bere T, et al. African Youth in Mind - Protocol of a Pilot feasibility trial of a brief psychological Intervention for older adolescents with depression delivered through senior high schools in Navrongo, Ghana. PLoS One. 2025;20(4):e0319462. doi: 10.1371/journal.pone.0319462 40168302 PMC11960926

[24] Doyle AM, Bandason T, Dauya E, McHugh G, Grundy C, Simms V, et al. Common mental health and emotional and behavioural disorders among adolescents and young adults in Harare and Mashonaland East, Zimbabwe: a population-based prevalence study. BMJ Open. 2023;13(3):e065276. doi: 10.1136/bmjopen-2022-065276 36918245 PMC10016291

[25] Lumivero. NVivo. 2017.

[26] Tong A, Sainsbury P, Craig J. Consolidated criteria for reporting qualitative research (COREQ): a 32-item checklist for interviews and focus groups. Int J Qual Health Care. 2007;19(6):349–57. doi: 10.1093/intqhc/mzm042 17872937

[27] Ibrahim A, Hor S, Bahar OS, Dwomoh D, McKay MM, Esena RK, et al. Pathways to psychiatric care for mental disorders: a retrospective study of patients seeking mental health services at a public psychiatric facility in Ghana. Int J Ment Health Syst. 2016;10:63. doi: 10.1186/s13033-016-0095-1 27729938 PMC5048657

[28] Badu E, Mitchell R, O’Brien AP. Pathways to mental health treatment in Ghana: Challenging biomedical methods from herbal- and faith-healing perspectives. Int J Soc Psychiatry. 2019;65(6):527–38. doi: 10.1177/0020764019862305 31277557

[29] Patel V, Simunyu E, Gwanzura F. The pathways to primary mental health care in high-density suburbs in Harare, Zimbabwe. Soc Psychiatry Psychiatr Epidemiol. 1997;32(2):97–103. doi: 10.1007/BF00788927 9050351

[30] Nefasi M. Exploring Pathways to Care in the Zimbabwean Mental Health Sector. IJRISS. 2024;VIII(II):2447–52. doi: 10.47772/ijriss.2024.802175

[31] Wasif M, Vargas M, Zrenchik K. Extended therapy sessions: sometimes more is better. 2024 [cited 2024 December 28]. Available from: https://allintherapyclinic.com/extended-therapy-sessions-sometimes-more-is-better/

[32] Read UM, Jilka S, Singh SP. Collaborating with Traditional and Faith Healers in Mental Health: A Public Health Approach. World Social Psychiatry. 2023;5(2):144–8. doi: 10.4103/wsp.wsp_28_23

[33] Hansen AS, Christoffersen CH, Telléus GK, Lauritsen MB. Referral patterns to outpatient child and adolescent mental health services and factors associated with referrals being rejected. A cross-sectional observational study. BMC Health Serv Res. 2021;21(1):1063. doi: 10.1186/s12913-021-07114-8 34625073 PMC8501731

[34] Udedi M. Improving access to mental health services in Malawi. 2016. Available from: https://www.researchgate.net/publication/10.13140/RG.2.1.3996.9524

[35] Hoeft TJ, Fortney JC, Patel V, Unützer J. Task-Sharing Approaches to Improve Mental Health Care in Rural and Other Low-Resource Settings: A Systematic Review. J Rural Health. 2018;34(1):48–62. doi: 10.1111/jrh.12229 28084667 PMC5509535

[36] Pine AE, Baumann MG, Modugno G, Compas BE. Parental involvement in adolescent psychological interventions: A meta-analysis. Clin Child Fam Psychol Rev. 2024. doi: 10.1007/s10567-024-00481-8 PMC11486598 38748300

[37] Saade S, Parent-Lamarche A, Khalaf T, Makke S, Legg A. What barriers could impede access to mental health services for children and adolescents in Africa? A scoping review. BMC Health Serv Res. 2023;23(1):348. doi: 10.1186/s12913-023-09294-x 37024835 PMC10080850

[38] Mongelli F, Georgakopoulos P, Pato MT. Challenges and Opportunities to Meet the Mental Health Needs of Underserved and Disenfranchised Populations in the United States. Focus (Am Psychiatr Publ). 2020;18(1):16–24. doi: 10.1176/appi.focus.20190028 32047393 PMC7011222

[39] Beacon Strategies. Streamlining Healthcare: The Role of Care Pathways. [cited 2024 December 27]. Available from: https://beaconstrategies.net/blog/the-role-of-care-pathways#:~:text=Care%20pathway%20%E2%80%94%20A%20representation%20of%20services%20and,and%20coordinated%20within%20a%20defined%20%27system%20of%20care%27

[40] Rotter T, Kinsman L, James E, Machotta A, Willis J, Snow P. The effects of clinical pathways on professional practice, patient outcomes, length of stay, and hospital costs: Cochrane systematic review and meta-analysis. Evaluation and the Health Professions. 2012;35:3–27. 10.1177/0163278711407313 21613244

