# Supplementary material for: The referral trajectories for young people with depression in low-resource settings: A qualitative study from Ghana and Zimbabwe
Source: PLoS One. 2026 Oct 5;21(10):e0358255. doi: 10.1371/journal.pone.0358255 (PMC13637860; doi:10.1371/journal.pone.0358255)
Supplement: Supplementary file 1 [file pone.0358255.s010.pdf]

Editor-in-Chief

PLOS ONE

Subject: Reviewer recommendation for manuscript PONE-D-25-35293

Dear Editor

Thank you for considering me to review the manuscript entitled: "... The Referral Trajectories for Young People with Depression in low resource settings: A Qualitative study from Ghana and Zimbabwe (Manuscript ID: PONE-D-25-35293).

The study explores an important topic in mental health, particularly for population considered vulnerable with complexities of resources. This study is timely, for addressing metal health care gaps in the treatment of young people. However, several areas require revision to strengthen methodological rigor, clarity, and coherence before publication.

The following areas require the attention of the authors:

Title and abstract

- **Title:** ensure that the word "low resource" is correctly written. It should be 'low-resource" instead of "low resource".
- **Abstract:** there is grammar and spelling errors that require the attention on the authors e.g., line 10 refers to **depressiom** instead of depression. Additionally, the abstract lacks methodological details that justify the 102 participants e.g. how that sampled, and the data saturation or adequacy was decided on. Keywords are few- the thumb rule for keywords is 6-8.

Methods

- **Design and theoretical framework:** It is unclear how the phenomenological theory was used in the context of a clear exploratory descriptive study. a phenomenological study focuses on lived experiences while exploratory qualitative research explored a specific phenomenon without consideration of the lived experiences, therefore even the analytical approach for a clear exploratory descriptive study would differ from a phenomenological study. see line 116-121 vs line 12. The authors are advised to clearly write this section. Perceptions cannot be understood through lived experienced, there is a clear discrepancy that needs to be clarified see line 119-121- "Phenomenological research focuses on participants lived experiences of a phenomenon by understanding their perceptions, feelings, and thoughts about the phenomenon and the meaning they attach to such experiences".

- **Study context:** it is not clear why the age varied in the different contexts even with the -current justification, there is a need to expand this section as it may have influenced comparability. If not, this should be expanded or detailed under limitations. See line 88-95.
- **Participant recruitment**: while the authors mention that the key informants were recommended, there is no mention of how they were approached and how informed consent was obtained.
- **Number of participants**: there is a discrepancy between the number of participants reported on the abstract vs the number of participants presented on Table 1 (102 vs 116), see line 13 vs line 142. It is recommended that the authors clarify the correct sample size.
- **Data collection:** while the manuscript reflects that an interview guide included a core list of open-ended questions. It does not reflect how they were formulated and pretested. Additionally, it does not reflect nature of questions asked. To correct this, please add the central question that was used, and a sample of the probing questions that were used since they were predetermined judging from the statement "The interview guides included a core list of open-ended questions and anticipated follow-up question" to interview. Translation in qualitative research is handled with careful considerations; how did the authors ensure that the meaning was maintained as there is no mention of backward translation?
- **Data analysis**: please manage the repeated headings under this section. There is no mention of the data analysis method used and the steps thereof. Ensure that this section is well described, include the method used to analyse the data, the role of the researchers during this process and describe if an independent qualitative data analyst was used , and how were the themes agreed upon?
- **Trustworthiness**- there is no section detailing how trustworthiness was ensured throughout the manuscript. This section is crucial in qualitative research. How did the authors ensure that the findings accurately reflect the perspectives of the participants?

Findings

- A thematic table would represent the results well, and further should who the themes and subthemes link. There figure 2 reflects trajectories of Ghana only, does it mean these are the same in Zimbabwe? The postulations made by the authors are worrisome for a qualitative study- what happens to bracketing our preconceived ideas particularly if the study is guided by the phenomenological framework? Postulating is therefore contradictory.

Discussion

- While the discussion is within a comparative approach, the discussion lack sufficient contrast or integration of the findings from the two contexts. It is therefore important to be deliberate about this, there is a need for the authors to compare and contrast the two contexts in depth and highlight country specific (such as barriers and enablers) – see discussion section, page 21-25.

Limitations

- This section does not capture the potential bias introduced in this study by postulating. It is also necessary to expand this section to include how the lack of voice of the youth might have influenced the findings, hence the need for further research.

Language and grammar

- There is a need for language editing to address grammatical and typographical errors. E.g., Depressiom instead of depression; widder instead of wider, etc.

Reference

- Please ensure that all references cited are on the list. Also ensure consistency on the list of references to adhere to the journal's required format.

Conclusion

Based on the above concerns, there are still areas that need to be addressed by the authors, I therefore recommend revision to strengthen methodological rigor and suitability for publication in PLOS ONE.
