# Supplementary material for: The referral trajectories for young people with depression in low-resource settings: A qualitative study from Ghana and Zimbabwe
Source: PLoS One. 2026 Oct 5;21(10):e0358255. doi: 10.1371/journal.pone.0358255 (PMC13637860; doi:10.1371/journal.pone.0358255)
Supplement: Supplementary file 3 [file pone.0358255.s002.docx]

**Interview Guide: Adolescents who have used the youth friendship bench**

**Introduction**

My name is…………….and I work for the Youth in Mind project. Thank-you so much for meeting me today. We have invited you to here so we can discuss what the mental health situation is like for young people in particular the problem of Kufungisisisa/ (depression and anxiety) as well talk about how we can best address these problems. We have also invited you because you have used Friendship Bench services and we would like to hear your experiences receiving care and how we can improve the service to meet your needs. There are no right or wrong answers, we genuinely want to hear your views so please feel free to be as honest as possible. The information will be used only for the purpose of the study and your confidentiality is assured.

[**Icebreaker**] Now to begin, please tell me a little bit about yourself and how you got to receive care at the Friendship?

**Explanatory model**

1. Please tell me, what is your understanding of mental health in particular Depression/ Kufungisisa?
2. How do other young people understand mental health? – what do they say it. What do they say causes poor mental health?
3. What are the most common mental health problems that young people experience/ face?
4. What do you think causes these mental health problems in young people?
5. Earlier we talked about different mental health problems that young people experience, what do you think needs to be done to address these problems?

**Stigma**

1. How do people normally react to someone who is having mental health problems (what do they say and how do they treat them)?
2. How do you think we must treat someone who is going through mental health problems (kufungisisa)?
3. How best can we prevent mental health related stigma?

**Experience receiving care at the Friendship Bench**

1. Please describe the care you received at the Friendship Bench [probe: content, delivery i.e., online, face to face, cadre of counsellor, what made you seek care]?
2. How many counselling sessions did you receive?
3. [If they received one probe reasons for failure to attend more? What can be done to ensure that people attend more sessions?
4. What did you find helpful about the Friendship Bench & why; what was not helpful and why]?
5. Can you tell us about your relationship with the counsellor [what did you like & what did you not like]?
6. What do you think can be done to improve the connection and make you more feel understood?
7. Did you join CKT [if yes, what was your experience like]?
8. How can we improve CKT to make it suit the needs of young people? [what type of activities can be included in CKT]
9. Do you still experience any of the issues and/or symptoms you mentioned earlier [give example of what symptoms they said they had had before FB like thinking too much / symptoms?]
10. What do you think needs to be improved/ changed in the FB to improve the care and meet your needs [probe, where it is provided, by whom, time of day, number of sessions, involve others ETC]
11. How do you think your parents or guardians should be involved in any mental health counselling? probe –, no involvement, information only, invite them to a meeting)

**Other sources of care sought.**

1. Did you try to get help elsewhere for any issues you described, before coming to the FB? [probe – where did you seek help?]
2. Can you describe your experience of getting help from that place/service / person?
3. How do you compare the care you received and the care you received at FB?
4. What elements of care you received there do you think we can integrate into FB?

**Barriers and enablers**

1. What usually gets in the way of young people in getting mental health care?
2. What would make it easier for young people to get help for mental health?
3. If you were given an opportunity to introduce a program that deals with depression in you people, what type of program would you introduce?
4. Where would this program be delivered (community clinic schools etc?
5. Do you think this should be provided one to one for the young person or do you think an intervention provided to a group?
6. Who do you think should provide the service?
7. How can young people be involved in running the project?
8. How can we involve parent in the program?
9. What other activities that give young people a sense of pleasure/ sense of happiness that we can incorporate into the program?

**Closing Question**

1. Is there anything else you would like to say about mental health that you feel we may have left out?
