# Supplementary material for: The referral trajectories for young people with depression in low-resource settings: A qualitative study from Ghana and Zimbabwe
Source: PLoS One. 2026 Oct 5;21(10):e0358255. doi: 10.1371/journal.pone.0358255 (PMC13637860; doi:10.1371/journal.pone.0358255)
Supplement: Supplementary file 4 [file pone.0358255.s003.docx]

**Village Health Workers – interview guide:**

1. General impressions: Tell me about how you first heard about the Friendship Bench?
   a. What were your first impressions of the Friendship Bench?
   b. Did you think it was needed in your community?
2. What is the goal of the Friendship Bench?
   a. What do you think about this goal?
3. Training: Tell me about how you were trained to deliver the Friendship Bench intervention. What was the training like?
   a. What happened first? What happened next?
   b. What was the best part of the training?
   c. What was the worst part of the training?
   d. Did you encounter situations with patients that were not covered in your training? Tell me about that.
4. Screening: While delivering the Friendship Bench intervention, what was the process for identifying people with mental illness?
   a. What did you do?
   b. Can you tell me about one patient who you screened?
   c. How did patients react to the screening? Can you tell me about a negative reaction from a patient?
5. Delivering the intervention: Can you tell me about one patient who you treated? What was that experience like? What did you do to treat that person?
   a. What was it like to deliver the Friendship Bench intervention?
   b. Tell me about a good outcome. What changed in the patient’s life?
   c. Can you tell me about a time when you deviated from problem solving therapy or the Friendship Bench in order to help a patient?
   d. Can you tell me about a time when you encountered a situation you felt like you could not handle? Or that was hard to handle?
      i. What was the hardest thing about delivering the intervention?
6. Supervision: What do you do when you feel like you have a challenging patient?
   a. Who do you look to for advice? Can you tell me about a time when you asked for advice?
   b. Is the level of support adequate? Too much? Too little?
   c. How do you know if you’re doing a good job? What happens if you are not?
   d. If you had an idea about how to make the Friendship Bench better who would you tell?
   e. What is it like to be supervised by nurses in the clinic?
7. Referral: Have you ever referred a patient out of the Friendship Bench?
8. Recruitment and retention
   a. What is your favorite part of your job?
   b. How did you balance the Friendship Bench with your other responsibilities?
   c. How do you see your role in the Friendship Bench going forward?
   d. How did being part of the Friendship Bench affect you emotionally? [Can say: “Treating people with mental illness can be emotionally challenging for many people” to normalize sharing difficult emotions. If it affected them emotionally, ask for an example of a time that was emotionally challenging.]
9. Suggestions: We want to improve the Friendship Bench in this community. What would you change to make the Friendship Bench better?
   a. What was the hardest part of working on the Friendship Bench?
10. What do you think will happen to the Friendship Bench in the future?
    a. What would have to change for the Friendship Bench to continue forever in this community?
11. Is there anything else that you think that we should know about the Friendship Bench?

**Nurses – interview guide**

1. Do you see patients who have depression or another mental illness?
   a. How do you know that they have mental illness?
   b. What do you do?
2. General Impressions: How did you first hear about the friendship bench?
   a. What were your first impressions of the friendship bench?
3. What is the goal of the friendship bench?
   a. What do you think about this goal?
4. Training: Tell me about how you were trained to supervise LHWs on the friendship bench. What was the training like?
   a. What happened first? What happened next?
   b. What was the best part of the training?
   c. What was the worst part of the training?
5. Did you encounter any situations that weren’t covered in training? Tell me about one of those. Tell me about another.
6. Supervising: What do you feel your role is in supervising LHWs?
   a. What is it like to supervise a group of LHWs?
   b. Tell me about a question or problem that a LHW has had on the Friendship Bench that the LHW came to talk to you about.
   c. How do you know if LHWs are doing a good job on the friendship bench?
   d. What happens if they are not doing a good job?
7. Receiving Referrals: What happens when a LHW refers a patient to you who needs a higher level of care? Walk me through that process.
   a. How do you know that a patient needs a higher level of care?
   b. What makes the process more difficult?
   c. What makes the process easier?
8. What role do you see yourself playing in the Friendship Bench going forward?
9. If you could change something about the Friendship Bench, what would it be?
10. Is there anything else that you think we should know about the Friendship Bench or your role in it?

**Patients – Interview guide**

1. Tell me about how you came to the Friendship Bench.
   a. How and why did you start going to the Friendship Bench?
   b. How did you get referred here? (Draw out the process)?
   c. Did you want to come?
2. What is your understanding of what the Friendship Bench is for?
   a. What is the goal?
3. Experience: What is it like using the Friendship Bench?
   a. How do you feel when you are talking with the LHW?
   b. What do you talk about? Can you give me an example of one thing that you discussed.
   c. How have you felt right before using the FB? Can you give me an example of a day that you felt that way?
   d. How have you felt right after? Can you give me an example of a day that you felt that way?
4. Can you tell me about a problem that you discussed at the Friendship Bench.
   a. Why was this troubling to you?
   b. What did the LHW say to you about this problem?
   c. What changed about this problem after you talked with the LHW?
5. Is there anything you would never discuss with the LHW?
6. What is the LHW like?
   a. What about her was helpful? (trying to get a sense of if she is compassionate, professional, good listener, trustworthy etc…)
   b. What about her wasn’t helpful?
7. General Impressions (After): How has your understanding of the friendship bench changed after using it?
   a. How did your impressions change after the first session?
   b. How did your impressions change after multiple sessions?
8. Access: How did you get to the Friendship Bench? Was it ever hard to get there when you needed it?
   a. Did you ever have to wait?
9. If you had a problem again, could you come back to the Friendship Bench? When would you do that? How would you do that?
10. Future Use: How do you see the friendship bench playing a role in your life going forward?
11. Communication: What do you tell other people about the Friendship Bench?
    a. Why do you explain it like that?
    b. How do people respond?
12. Attitudes toward CMDs: What does the word [mental health][depression][anxiety][kufungisisa] mean to you?
13. If you could change something about the friendship bench what would it be?
14. Is there anything else that you think that we should know about the Friendship Bench?

**Community/family members – interview guide**

1. From your perspective, what is the Friendship Bench?
   a. What is it for?
   b. Why do people go there?
   c. What type of people go there?
2. How did you hear about the Friendship Bench?
3. Do you know anyone who has gone to the Friendship Bench? Tell me about that person.
   a. Why did they go?
   b. What was that like for them?
   c. What happened to them afterwards? Did anything change?
   d. How has this affected you?
4. Do you know anyone who hasn’t gone to the Friendship Bench, but should? Tell me about that person.
   a. Why should they go?
   b. Why would the Friendship Bench help them?
5. How do you explain the Friendship Bench to people who don’t know what it is?
   a. Why do you explain it that way?
6. Broader Effects on the Community: How has the Friendship Bench affected your family (or community if they do not have a family member who attended)?
   a. How does your family/community talk about the Friendship Bench?
7. What happens when someone gets a mental illness?
   a. What happens to that person?
   b. How do they get the illness?
8. If you could change something about the Friendship Bench, what would it be?
9. Is there anything else you think that we should know about your community and the Friendship Bench?
