# Supplementary material for: The referral trajectories for young people with depression in low-resource settings: A qualitative study from Ghana and Zimbabwe
Source: PLoS One. 2026 Oct 5;21(10):e0358255. doi: 10.1371/journal.pone.0358255 (PMC13637860; doi:10.1371/journal.pone.0358255)
Supplement: Supplementary file 5 [file pone.0358255.s004.docx]

**Interview Guide: High School Students**

**Introduction**

My name is…………….and I work for the Youth in Mind project. Thank-you so much for meeting me today. As a student here we have invited you to here so we can discuss what the mental health situation is like for young people in particular the problem of Kufungisisisa/ depression both in and outside school. It is ourt hope that the information you are going to share with us is will help us develop mental health intervention for young people like yourself who may be experiencing depression. There are no right or wrong answers, we genuinely want to hear your views so please feel free to be as honest as possible. The information will be used only for the purpose of the study and your confidentiality is assured.

**Icebreaker**

1. Kindly tell me about yourself and the course you study at school?

**Explanatory model**

1. Please tell me what your understanding of mental health is?
2. What do other young people think about mental health?
3. What are the most common issues related to depression that young people experience?
4. What do you think causes depressions in young people?

**Stigma**

1. How do people normally react to someone who is going through depression or having mental health problems.
2. How do you think they ought to be treated?
3. How best can we reduce mental health stigma?

**Barriers and enablers**

1. What usually gets in the way of young people getting mental health care for depression?
2. What would make it easier for young people to get help for depression?

**Current care provided at school**

1. Can you please tell me about the mental health services provided here at school for depression ? [probe_1: who provides care, where, how? Probe_2: psychosocial services: who provides care, where, how?)
2. What do you think about the current care offered for derpressions? [probe what works well, what does not work well and why?
3. In your view, what do you think are the missing gaps (what is the care not doing, that it should be doing) in the provision of care for depressionre services in high schools in general?  How can these gaps be overcome?

**Potential Intervention**

1. If we were to introduce an intervention for managing depression for young people in high schools, what type of intervention would you like this to be?
2. What kind of issues do you think the intervention can try to address?
3. Do you think this should be provided one to one or do you think an intervention provided to a group of students all experiencing stress would be more helpful or more suitable?
4. Who do you think will be best suited to deliver the intervention?
5. What do you think about another young person / peer’s ability to provide counselling to you?
6. Where would you like the treatment to be delivered? [Probe school, online, another location, please describe]
7. Would you attend treatment if delivered at the school?
8. How should the student’s parents or guardians be involved in any mental health counselling? probe –, no involvement, information only, invite them to a meeting, other

**Appreciation:**

Our discussion/interview has come to an end, on behalf of the PI (**Professor Chibanda**) and the rest of the team, I wish to thank you for your participation and to assure you again that the information collected will be treated confidentially and used only for the purposes of the study. Your identity will also be protected in all aspects of the study. Feel free to contact us through the telephone numbers provided on the informed consent form if you have any concerns and will like to discuss it with us.  I hope you will be willing to participate in future studies. Farewell
