# Supplementary material for: The referral trajectories for young people with depression in low-resource settings: A qualitative study from Ghana and Zimbabwe
Source: PLoS One. 2026 Oct 5;21(10):e0358255. doi: 10.1371/journal.pone.0358255 (PMC13637860; doi:10.1371/journal.pone.0358255)
Supplement: Supplementary file 6 [file pone.0358255.s005.docx]

**INTERVIEW GUIDE: FB Counsellors**

**Introduction**

My name is…………….and I work for the Youth in Mind project.  Thank-you so much for meeting me today. We have invited you to here so we can discuss what the mental health situation is like for young people in particular the problem of Kufungisisisa/ depression as well talk about how we can best address these problems. We have also invited you because you have work on the Friendship Bench as a counsellor, and we would like to hear your experiences receiving care and how we can improve the service to meet your needs. There are no right or wrong answers, we genuinely want to hear your views so please feel free to be as honest as possible. The information will be used only for the purpose of the study and your confidentiality is assured.  .

[**Icebreaker**] Can you please tell me about yourself and how you ended up as an FB counsellor?

**Explanatory model**

1. How do young people think about mental health ?
2. What are the most common mental health problems that young people experience?
3. What do you think causes these mental health problems in young people?

**Stigma**

1. How do people normally react to someone who is going through depression?
2. How best can we address this mental health stigma?

**Delivering PST face to face**

1. Please describe your experience of providing mental health care for young people [Probe: What do you find interesting, what do you find difficult]
2. What are the common issues that young people 15-24 bring to the Friendship Bench?
3. Do you feel the friendship bench sessions are sufficient to address all problems that young people present with? If no, which problems cannot be solved through PST? How can these problems be solved?
4. What components of PST do you find useful for young people?
5. What challenges do you face when implementing action plans with young people?
6. How can we overcome these challenges?

**Barriers to retention**

7. What do you think are the main challenges in getting young people to start therapy?
8. For young people who have started therapy, why do you think they do not come back for more sessions? [probe: can you please describe challenges, Probe: financial, : distance, transport money, lactating mothers, employed participants pressed for time, disclosure, household commitments, other work, physical mobility problems, not disclosed to family, felt unsafe/at risk of violence]
9. What do you think should be changed so that young people come back for more a) face to face sessions and b) online sessions?
10. From your experience, do you feel more sessions should be offered for young people presenting with complex problems. Can you explain more?

**Online Therapy Delivery via Open line and WhatsApp**

11. What has been your experience like delivering PST online either via WhatsApp or Inuka ?
12. What features of the online counselling help you conduct sessions effectively? Are there any specific features you find most helpful and why?
13. Can you describe any challenges you experienced in getting young people to do online sessions?
14. Do you have any other advice for us about how we can improve the online experience for both you and the clients?
15. Would you recommend the online counselling or face to face sessions to someone? Please explain more?

**Referral**

16. What type of referral pathways are currently available for young people and their issues? Can you name some of these referral places that you know?
17. What kind of referral pathways need to put in place for young people?

**Training**

18. Do you feel the training prepared you adequately to provide therapy to young people [**probe: in what ways**]?
19. Are there any problems during counselling that you find difficult to deal with?
20. What kind of training or support would you need in order to better handle these cases?

**Supervision and Support**

21. Support and supervision – is the support you receive appropriate and sufficient [probe: support from supervisors, management, rest of the staff]?
22. In what ways could we improve the supervision to help you to support young people who bring different issues to the sessions [E.g., substance use, suicidal thinking, abuse, family, interpersonal issues)?

**Other potential intervention strategies**

1. What other components or activities do you think can be added to the Friendship Bench that will help it cater for the needs of young people with kufungisisisa? (activities that give young people a sense of pleasure/ happiness)
