# Supplementary material for: The referral trajectories for young people with depression in low-resource settings: A qualitative study from Ghana and Zimbabwe
Source: PLoS One. 2026 Oct 5;21(10):e0358255. doi: 10.1371/journal.pone.0358255 (PMC13637860; doi:10.1371/journal.pone.0358255)
Supplement: Supplementary file 7 [file pone.0358255.s006.docx]

**Interview Guide: Teachers/School Heads**

**Introduction**

My name is…………….and staff of the Youth in Mind project at Navrongo Health Research Centre. As explained to you earlier on during the consenting, you have been invited to participate in this interview because you are a School Head, School Teacher, or School Focal person. The purpose of this this interview is to understand your views and experiences when it comes to mental health among students, barriers to the provision of care for students and potential intervention strategies. The information that you will volunteer in this study will enable us to develop a Mental Health intervention for students in senior high school (15-18 years). I wish to request you give honest responses to the questions as much as possible. The information will be used only for the purpose of the study and your confidentiality is assured.

**Theme 1: Nature of common mental health conditions**

**Information we are looking for:**

- Knowledge about depression/anxiety among Teachers/school heads

1. What are the common mental health issues faced by students in your school?
2. Kindly tell me about the symptoms they usually present.
3. What is your understanding of depression and anxiety [EMOTIONAL DIFFICULTIES]?
4. How would you know that your student is experiencing depression and anxiety [EMOTIONAL DIFFICULTIES]?
5. Are there specific groups of students that are at greater risk of having an issue with depression and anxiety [emotional difficulties] (i.e. boys vs girls, pregnant girls, HIV positive, lack of father, household income, use and or abuse of alcohol and other substances)? Why is that the case?
6. What do you think causes depression and anxiety [emotional difficulties] among students? Probe for the most common causes including stigma by teachers, school authorities, and peers)

**Theme 2: School mental health services, programmes, and policies**

**Information we are looking for:**

- Existing mental health services in schools
- Suitability of the school environment in delivering mental health services
- Availability of mental health professionals and referral system
- Barriers to delivering mental health services

7. What services are available in your school to help students with depression and anxiety, [emotional difficulties] [probe who provides care, where and how, involvement of parents]
8. In your experience how suitable is the school environment for delivering mental health services to students? *Explain.*
   a. What guidelines are available to guide the provision of mental healthcare services to students? Are these guidelines recognized nationally?)  To what extent are these used?
   b. What guidelines are in place for referring students **from schools to health facilities for care?** *Please provide examples if possible.* How functional is the referral system?
9. What are your thoughts on the help that is currently offered to students when they experience depression and anxiety [emotional difficulties] (probe for reasons - what has worked well in the past to help students who are having depression and anxiety [emotional difficulties]? What hasn’t worked well)
10. What do you think can be done to make the care offered relevant to students? Probe: what of making it appealing?
11. What do you think are the main challenges in the provision of care to help students experiencing depression and anxiety [emotional difficulties] (probe – time, human resources, training, knowledge, etc, availability of medicines)?
12. How do you feel about students who experience depression and anxiety [emotional difficulties] (probe – for stigma, etc)

**Theme 3: Barriers and enablers mental health intervention**

**What we want to know**

- Enablers to mental health seeking
- Barriers to mental health seeking

13. What do you think will be some of the challenges to implementing depression and anxiety [emotional difficulties] intervention in schools? [probe- policies, time, human resources, adequate training, knowledge, availability of medicines, stigma]
14. What do you think would make it easier to implement the interventions in schools?
15. Any final thoughts about how we could make the intervention attractive to students?

**Theme 4: Intervention content, acceptability and feasibility**

**Information we are looking for:**

- Perception about the acceptability of Y-MIND intervention for students
- Expectations about the content of the intervention and how it could be delivered

We are thinking about developing a programme to support students with depression and anxiety [Emotional difficulties] who are attending secondary schools in the Kassena-Nankana Districts. We plan to first provide counselling to these students, and if that is not working, we will let a mental health professional attend to them.

16. How useful will it be to introduce this intervention for the treatment of depression and/or anxiety among students in Senior High School? How suitable is your school environment for this intervention? *Probe about suitability of available infrastructure.*
17. If this intervention were to be introduced, what do you think it should include to make it relevant to students (i.e appropriate)? *Why? What would make it appealing (attractive)? Why?*
18. Who should be involved in delivering this intervention? [ probe: peers of the same age group, male/female, teacher, school counsellor, school nurse?] Please give reasons
19. How should this be provided [Probe: one-on-one or provided to a group of students all experiencing depression and anxiety [emotional difficulties]? Why?
20. Where [location] do you think is the best place to deliver the intervention? Why? Probes: at school? outside school if so, where?
21. When would be the best time of the day to deliver the intervention in the school? Why?
22. How should we involve the student’s parents or guardians in our mental health intervention? Why?
23. Aside counselling, what other school activities can be put in place to help a student deal with depression and anxiety [emotional difficulties]?
24. What do you think about mental health care counselling services being delivered online such as WhatsApp compared to face-to-face?

**Appreciation:**

Our discussion/interview has come to an end, on behalf of the Country Lead (**Dr Benedict Weobong**) and the rest of the team, I wish to thank you for your participation and to assure you again that the information collected will be treated confidentially and used only for the purposes of the study. Your identity will also be protected in all aspects of the study. Feel free to contact us through the telephone numbers provided on the informed consent form if you have any concerns and will like to discuss them with us.  I hope you will be willing to participate in future studies. Thank You!

**FGD/IDI session summary form**

(To be completed by both moderator/interviewer and note-taker immediately after the session)

1. Date FGD/IDI conducted:
2. Moderator/interviewer:
3. Note-taker(s):
4. Participants (list)/Respondent code:
5. Duration of FGD/IDI:

Based on your experience of this FGD, please answer the questions below:

1. How would you describe the atmosphere and context of the discussion/interview?
2. What were the main points made during this discussion/interview?
3. What things (about participants, responses, new information, questions you were asked or had to clarify) surprised you?
4. What do you think about the quality of the data gathered in this session? Why?
5. Were there any problems with the topic guide (e.g. wording, order of topics, missing topics) you experienced in this session?
6. What messages did you take from this interview for intervention design?
7. Any other remarks or observations
