# Supplementary material for: The referral trajectories for young people with depression in low-resource settings: A qualitative study from Ghana and Zimbabwe
Source: PLoS One. 2026 Oct 5;21(10):e0358255. doi: 10.1371/journal.pone.0358255 (PMC13637860; doi:10.1371/journal.pone.0358255)
Supplement: Supplementary file 8 [file pone.0358255.s007.docx]

**Interview Guide: Ministry of Health/Ghana Health Service Officials**

**Interview process:** Interviews should be tailored to the respondents’ area of expertise. The key informant should have been sent the questions below in advance so that they could have the chance to prepare for the interview.

**Introduction**

My name is……………., and a staff of the Youth in Mind project at Navrongo Research Health Centre. As explained earlier during the consenting process, you have been invited to participate in this interview because you are a District Health Promoter/Ministry Mental Health Focal Person. The purpose of this interview is to understand your views and experience on mental health for your people, barriers to the provision of care and potential intervention strategies. The information that you will volunteer in this study will enable us to develop a Mental Health intervention for adolescents (15-18 years). I wish to request you give honest responses to the questions as much as possible. The information will be used only for the purposes of the study and your confidentiality is assured.

**Introduction**

1. Kindly tell me about your profession and what you do?
2. How long have you been working in this capacity?

**Theme 1: Adolescent health services, programmes, and policies**

**Information we are looking for:**

- Organisation of adolescent health services in the district
- Adolescent health programmes and policies (School Health Education Programme)

3. What health care services are provided to adolescents in the Navrongo area? Can you provide examples of the types of services provided? Please also elaborate on where these services are most often offered (such as in schools, health facilities, through outreach services, etc.). What organizations provide each service? **[If they have not mentioned a school health service, ask: “Is there a school health programme in the Navrongo area? If yes, explore further eg. What is included? What organization(s) are involved and for which aspects?]**
4. Would you say that these services are offered equally across groups of adolescents (such as adolescents from high socioeconomic status (SES) families vs. low SES, boys vs. girls or older vs. younger adolescents)? *If there are discrepancies, can you elaborate on why they exist?*
5. What are your thoughts on these services – do you think these are useful? Do you think they are suitable for the population?
6. What systems are there for referring adolescents **from primary care to a specialist?** *Please provide examples if possible*. How functional are they?
7. What systems are in place for referring adolescents **from schools to primary care or to a specialist?** *Please provide examples if possible.* How functional are they?
8. Do adolescents have to pay out-of-pocket for all the health services that they receive? If not, which ones are free? And which are covered by health insurance?
9. What guidelines or protocols are available to guide the provision of healthcare services to young people?
10. Based on your knowledge to what extent have these guidelines been implemented in routine health care? What do you think needs to be done to ensure that they are implemented fully?

**Theme 2: Intervention content, acceptability and feasibility**

**Information we are looking for:**

- Perception about the acceptability of Y-MIND intervention by adolescents
- Expectations about the content of the intervention and how it could be delivered

We are planning a stepped-care intervention to treat depression and/or anxiety among adolescents in secondary schools in the Navrongo area. This comprises brief psychological therapy (problem-solving therapy), with the option of adding an antidepressant for those who do not recover

11. We are planning to test whether introducing a mental health intervention for the treatment of depression and/or anxiety among adolescents in Senior High Schoool would be a good idea. What do you think about this?

*Explore.*

12. If this intervention were to be introduced, what do you think it should include to make it relevant and appealing to adolescents? *Why? Explore*
13. Which of these interventions would you prefer and why: delivering “coping with depression and anxiety [EMOTIONAL DIFICULTIES]” workshops in a large group of young people, a small group of young people, or one-to-one?
14. What do you think about mental health care counselling services being delivered online/WhatsApp as compared to face-to-face?
15. Who should be involved in delivering this intervention? (probe why and what sort of training would they need).What do you think about mental health care counselling services being delivered online/WhatsApp as compared to face-to-face?

**Theme 3: Barriers and enablers**

**What we want to know**

- Enablers to health seeking
- Barriers to health seeking

16. In your view, what do you think will be some of the barriers to implementing a mental health intervention for depression and anxiety [emotional difficulties] in schools? [ probe- stigma, policies, time, human resources, adequate training, knowledge, availability of medicines, stigma]
17. Are there any groups of young people that may experience specific barriers to receiving mental health treatment (i.e. younger vs older, boys vs girls, young people with specific issues at home) and why?
18. What can be done to make the intervention relevant and appealing to young people? (probe why)

**Appreciation:**

Our discussion/interview has come to an end, on behalf of the Country Lead (**Dr Benedict Weobong**) and the rest of the team, I wish to thank you for your participation and to assure you again that the information collected will be treated confidentially and used only for the purposes of the study. Your identity will also be protected in all aspects of the study. Feel free to contact us through the telephone numbers provided on the informed consent form if you have any concerns and will like to discuss it with us. I hope you will be willing to participate in future studies. Thank you!

**FGD/IDI session summary form**

(To be completed by both moderator/interviewer and note-taker immediately after the session)

1. Date FGD/IDI conducted:
2. Moderator/interviewer:
3. Note-taker(s):
4. Participants (list)/Respondent code:
5. Duration of FGD/IDI:

Based on your experience of this FGD, please answer the questions below:

1. How would you describe the atmosphere and context of the discussion/interview?
2. What were the main points made during this discussion/interview?
3. What things (about participants, responses, new information, questions you were asked or had to clarify) surprised you?
4. What do you think about the quality of the data gathered in this session? Why?
5. Were there any problems with the topic guide (e.g. wording, order of topics, missing topics) you experienced in this session?
6. What KEY messages did you take from this interview for intervention design?
7. Any other remarks or observations
