# Supplementary material for: The referral trajectories for young people with depression in low-resource settings: A qualitative study from Ghana and Zimbabwe
Source: PLoS One. 2026 Oct 5;21(10):e0358255. doi: 10.1371/journal.pone.0358255 (PMC13637860; doi:10.1371/journal.pone.0358255)
Supplement: Supplementary file 9 [file pone.0358255.s008.docx]

**Interview Guide: Adolescents WITH MENTAL HEALTH HISTORY**

**Introduction**

My name is…………….and I work for the Youth in Mind project at Navrongo Health and Research Centre. We have invited you here so we can discuss what the mental health situation is like for young people in particular the problem of depression and anxiety [Emotional difficulties] as well as talk about how we can best address these problems. We have also invited you because you have used mental health services and we would like to hear about your experiences receiving care and how we can improve the service to meet your needs. There are no right or wrong answers, we genuinely want to hear your views so please feel free.

[**Icebreaker**] Now to begin, can you please tell me a little bit about yourself [where you are from what you do etc]?

**Theme 1: Explanatory model**

**Information we are looking for**

- Adolescents’ beliefs about mental illness

1. Please tell me what you understand about mental health or mental illness?
2. How do other young people think about mental health or mental illness?
3. What are the most common mental health problems that young people experience?
4. What do you think causes these mental health problems in young people?

**Theme 2: Experiences**

**Information we are looking for**

- Adolescents ‘experiences of mental health conditions
- Experience of stigma
- Coping strategies
- Mental health care received

5. Tell me what led you to seek mental health care services? (Probe for symptoms; thinking too much, sleep issues, etc). Which of these symptoms or experiences do you find it challenging to deal with?
6. What kinds of things do you do to help deal with the mental health issue affecting you? Do you get help from outside family or friends? Who was able to help you?” (probes – “someone at school/community? A priest/mallam/traditionalist? A Dr? Or from a Primary Health Centre? Or a self-help group?
7. For the mental health issue that you had, where did you first seek help from? What was the experience like? **[IF HEALTH FACILITY MENTIONED, SKIP TO QUESTION 8 IF NOT SKIP TO QUESTION 11]**
8. For the mental health issue that you had, did you seek help from a health facility?
9. How was the mental health care service delivered to you at the health facility? (Probe: content, delivery i.e. online, face to face, group, the cadre of counsellor)
10. What did you find helpful about the care offered by the health facility (probe for examples/ in what ways) (probe for what content/approaches were helpful?
11. What did you not find helpful about the care offered by the health facility (probe for examples) (probe for what content/approaches were not helpful?
12. How concerned were you about what your friends/family/teachers/community members thought about you receiving treatment? (Probe for stigma)

**Theme 3: Persistent symptoms/ difficulties after getting mental health care**

**What we want to know:**

- Coping strategies for persistent symptoms

13. Do you still experience any of the issues you mentioned earlier (give an example of what symptoms they said they had had before, for example thinking too much / symptoms?) **IF THE ADOLESCENT NO LONGER EXPERIENCE SYMPTOMS SKIP TO THE NEXT SECTION**
14. Have you tried to get help elsewhere for any issues/symptoms you are still having? probe – where did you seek help? What was your experience of getting help from that place/service/person?

**Theme 4: Behavioural activation**

**Information we are looking for:**

- Activities adolescents engage in that makes them feel a sense of pleasure or happiness
- Activities adolescents engage in that makes them feel a sense of achievement

15. Are there any activities or things that you do for yourself that makes you have a sense of pleasure or happiness? (Probe for: taking a walk, listening to music, watching movie, singing, exercise, meditation, etc)
16. What activities give you a sense of achievement or satisfaction in your life? (Probe for: activities)
17. What activities give you a sense of feeling connected or close to other important people in your life, such as your friends or family?

**Theme 5: Barriers and enablers**

**What we want to know**

- Enablers to mental health seeking
- Barriers to mental health seeking

18. Did you face any challenges in your quest to seek mental health care services? If yes, can you kindly tell me about the challenges you faced? If not, what are some of the challenges you think will get in the way of adolescents seeking mental health care?
19. What would make it easier for young people to get help for mental health?

**Theme 6: Intervention content, expectations, and mode of delivery**

**Information we are looking for:**

- Adolescents with MH history’s perception about the acceptability of Y-MIND intervention by adolescents
- Expectations about the content of the intervention and how it could be delivered

We are thinking about developing a programme to support young people with depression and anxiety [Emotional difficulties] who are attending secondary schools in the Navrongo area. We plan to first provide counselling to these adolescents, and if that is not working, we will let a mental health professional attend to them.

20. What do you think about this intervention? (Probe for acceptability among young people)
21. WHO should deliver the counselling? [probe: peers of the same age group, male/female, teacher, school counsellor, school nurse?] Please give reasons
22. What do you think about mental health care counselling services being delivered online/WhatsApp as compared to face-to-face?
23. In your opinion, how difficult will it be to trust your online counsellor?
24. How could an online counsellor help build trust with the you?
25. Aside from talking about their issues, what other activities can be put in place to help a you deal with depression and anxiety?

**Appreciation:**

Our discussion/interview has come to an end, on behalf of the Country Lead (**Dr Benedict Weobong**) and the rest of the team, I wish to thank you for your participation and to assure you again that the information collected will be treated confidentially and used only for the purposes of the study. Your identity will also be protected in all aspects of the study. Feel free to contact us through the telephone numbers provided on the informed consent form if you have any concerns and will like to discuss it with us. I hope you will be willing to participate in future studies. Thank you!

**FGD/IDI session summary form**

(To be completed by both moderator/interviewer and note-taker immediately after the session)

1. Date FGD/IDI conducted:
2. Moderator/interviewer:
3. Note-taker(s):
4. Participants (list)/Respondent code:
5. Duration of FGD/IDI:

Based on your experience of this FGD, please answer the questions below:

1. How would you describe the atmosphere and context of the discussion/interview?
2. What were the main points made during this discussion/interview?
3. What things (about participants, responses, new information, questions you were asked or had to clarify) surprised you?
4. What do you think about the quality of the data gathered in this session? Why?
5. Were there any problems with the interview guide (e.g. wording, order of topics, missing topics) you experienced in this session?
6. What **KEY** messages did you take from this interview for intervention design?
7. Any other remarks or observations
