# Supplementary material for: The referral trajectories for young people with depression in low-resource settings: A qualitative study from Ghana and Zimbabwe
Source: PLoS One. 2026 Oct 5;21(10):e0358255. doi: 10.1371/journal.pone.0358255 (PMC13637860; doi:10.1371/journal.pone.0358255)
Supplement: Supplementary file 10 [file pone.0358255.s009.docx]

**Interview guide for Mental Health Care Service Providers**

**Introduction**

My name is…………….and a staff of the Youth in Mind project in Navrongo Health Research Centre. As explained to you earlier on during the consenting, you have been invited to participate in this interview because you have experience of delivering mental health care services. The information that you will provide in this study will enable us to develop a Mental Health intervention for students (15-18 years). The information will be used only for the purposes of the study and your confidentiality is assured as such I would like to request that you give honest responses as much as possible.

**Ice breaker/orientation questions:**

a. How has been you’re your day?

**Background characteristics of key informants**

| Age |  |
| --- | --- |
| Sex |  |
| Religion |  |
| Highest Level of Education |  |
| Ethnicity |  |
| Profession |  |
| Current position |  |
| Years of service in current position |  |

**Theme 1: Nature of common mental health conditions**

**Information we are looking for:**

- Common mental health conditions and existing interventions
- Knowledge and practice of Problem Solving Therapy (PST)
- Knowledge about depression/anxiety among students

1. Please describe your experience of providing mental health care for young people [Probe: What do you find interesting? What do you find difficult **(Probe for specific symptoms or behaviours)**, how enjoyable is the work? How rewarding is the work.
2. What kind of mental health issues do students come here with?. **[which one is the commonest?]**
3. What are some of the services you provide for students when they are seeking mental health care services? [probe: for Problem Solving Therapy and for counselling and Advice/any other types of services]
4. Please tell me about your experience in using PST in your work with students (what do you think is the right approach – PST, behavioural activation? **[IF RESPONDENT HAS NO EXPERIENCE WITH PST, ASK ABOUT WHAT PSYCHOLOGICAL TREATMENT APPROACH THEY USE]**
5. What do you think about the ability of the health system to address all mental health problems that students present with? which problems cannot be solved through existing health services? How can these problems be solved?
6. Can you tell me about some of the challenges you face in the provision of mental health care services?

**Theme 2: Intervention content, expectations, and mode of delivery**

**Information we are looking for:**

- Service Providers’ perception about acceptability of Y-MIND intervention by students
- Expectations about content of intervention and how it could be delivered

We are planning a stepped care intervention to treat depression and/or anxiety among students in secondary schools in the Kassena-Nankana Districts. This comprises brief psychological therapy (problem solving therapy), with the option of adding an antidepressant for those who do not recover.

7. Based on your experience, what would be the attitude of students to this intervention? [Probe: for acceptability – useful/helpful]
8. Based on your experience of the needs of students, what do you think should be included in this intervention to make it effective?
9. WHO do you think should deliver the various steps of the intervention? [Probe: peers of the same age group, male/female teacher, school counsellor, school nurse?] please give reasons
10. HOW should the intervention be delivered? [Probe: one-on-one with the student or group etc.]
11. What kind of referral pathways need to be put in place for students with emotional difficulties (depression and/or anxiety) in school?
12. What kind of challenges are we likely to encounter with delivering this intervention?
13. What do you think about using online applications like WhatsApp for delivering therapy?

**Theme 3: Barriers and enablers**

**What we want to know**

- Enablers to mental health seeking
- Barriers to mental health seeking

9. What difficulties do you think students will face with attending online sessions on WhatsApp?  [Probe: stigma, financial, distance, transport money, lactating mothers, , disclosure, household commitments, other work, physical mobility problems, not disclosed to family, felt unsafe/at risk of violence]
10. What do you think will make this intervention attractive to young people? Is there anything we should do to make it more attractive to young people with depression/ anxiety?

**Appreciation:**

Our discussion/interview has come to an end, on behalf of the Country Lead (**Dr Benedict Weobong**) and the rest of the team, I wish to thank you for your participation and to assure you again that the information collected will be treated confidentially and used only for the purposes of the study. Your identity will also be protected in all aspects of the study. Feel free to contact us through the telephone numbers provided on the informed consent form if you have any concerns and will like to discuss it with us. I hope you will be willing to participate in future studies. Thank you!

**FGD/IDI session summary form**

(To be completed by both moderator/interviewer and note-taker immediately after the session)

1. Date FGD/IDI conducted:
2. Moderator/interviewer:
3. Note-taker(s):
4. Participants (list)/Respondent code:
5. Duration of FGD/IDI:

Based on your experience of this FGD, please answer the questions below:

1. How would you describe the atmosphere and context of the discussion/interview?
2. What were the main points made during this discussion/interview?
3. What things (about participants, responses, new information, questions you were asked or had to clarify) surprised you?
4. What do you think about the quality of the data gathered in this session? Why?
5. Were there any problems with the topic guide (e.g. wording, order of topics, missing topics) you experienced in this session?
6. What KEY messages did you take from this interview for intervention design?
7. Any other remarks or observations
