# Supplementary material for: The referral trajectories for young people with depression in low-resource settings: A qualitative study from Ghana and Zimbabwe
Source: PLoS One. 2026 Oct 5;21(10):e0358255. doi: 10.1371/journal.pone.0358255 (PMC13637860; doi:10.1371/journal.pone.0358255)
Supplement: Peer review history

# Peer review: Decision Letter 0

**DOI:** 10.1371/journal.pone.0358255.r001

**Authors:** Sogo France Matlala

22 Dec 2025

Dear Dr. Makiyi,

Thank you for submitting your manuscript to PLOS ONE. After careful consideration, we feel that it has merit but does not fully meet PLOS ONE’s publication criteria as it currently stands. Therefore, we invite you to submit a revised version of the manuscript that addresses the points raised during the review process.

Please address the important comments raised by Reviewer 1.

Please submit your revised manuscript by Feb 05 2026 11:59PM. If you will need more time than this to complete your revisions, please reply to this message or contact the journal office at plosone@plos.org. When you're ready to submit your revision, log on to https://www.editorialmanager.com/pone/ and select the 'Submissions Needing Revision' folder to locate your manuscript file.

- A letter that responds to each point raised by the academic editor and reviewer(s). You should upload this letter as a separate file labeled 'Response to Reviewers'.
- A marked-up copy of your manuscript that highlights changes made to the original version. You should upload this as a separate file labeled 'Revised Manuscript with Track Changes'.
- An unmarked version of your revised paper without tracked changes. You should upload this as a separate file labeled 'Manuscript'.

If you would like to make changes to your financial disclosure, please include your updated statement in your cover letter. Guidelines for resubmitting your figure files are available below the reviewer comments at the end of this letter.

If applicable, we recommend that you deposit your laboratory protocols in protocols.io to enhance the reproducibility of your results. Protocols.io assigns your protocol its own identifier (DOI) so that it can be cited independently in the future. For instructions see: https://journals.plos.org/plosone/s/submission-guidelines#loc-laboratory-protocols. Additionally, PLOS ONE offers an option for publishing peer-reviewed Lab Protocol articles, which describe protocols hosted on protocols.io. Read more information on sharing protocols at https://plos.org/protocols?utm_medium=editorial-email&utm_source=authorletters&utm_campaign=protocols.

We look forward to receiving your revised manuscript.

Kind regards,

Sogo France Matlala, PhD

Academic Editor

PLOS One

Journal Requirements:

When submitting your revision, we need you to address these additional requirements.

1. Please ensure that your manuscript meets PLOS ONE's style requirements, including those for file naming. The PLOS ONE style templates can be found at

https://journals.plos.org/plosone/s/file?id=wjVg/PLOSOne_formatting_sample_main_body.pdf and

https://journals.plos.org/plosone/s/file?id=ba62/PLOSOne_formatting_sample_title_authors_affiliations.pdf

2. When you resubmit, please ensure that you provide the correct grant numbers for the awards you received for your study in the ‘Funding Information’ section.

Please include a complete copy of PLOS’ questionnaire on inclusivity in global research in your revised manuscript. Our policy for research in this area aims to improve transparency in the reporting of research performed outside of researchers’ own country or community. The policy applies to researchers who have travelled to a different country to conduct research, research with Indigenous populations or their lands, and research on cultural artefacts. The questionnaire can also be requested at the journal’s discretion for any other submissions, even if these conditions are not met.  Please find more information on the policy and a link to download a blank copy of the questionnaire here: https://journals.plos.org/plosone/s/best-practices-in-research-reporting. Please upload a completed version of your questionnaire as Supporting Information when you resubmit your manuscript.

3. We note that the grant information you provided in the ‘Funding Information’ and ‘Financial Disclosure’ sections do not match.

4. Thank you for stating the following financial disclosure:

“The Youth in Mind consortium is a National Institute for Health Research (NIHR)-funded project (NIHR 133384)”

Please state what role the funders took in the study.  If the funders had no role, please state: "The funders had no role in study design, data collection and analysis, decision to publish, or preparation of the manuscript."

If this statement is not correct you must amend it as needed.

Please include this amended Role of Funder statement in your cover letter; we will change the online submission form on your behalf.

5. When completing the data availability statement of the submission form, you indicated that you will make your data available on acceptance. We strongly recommend all authors decide on a data sharing plan before acceptance, as the process can be lengthy and hold up publication timelines. Please note that, though access restrictions are acceptable now, your entire data will need to be made freely accessible if your manuscript is accepted for publication. This policy applies to all data except where public deposition would breach compliance with the protocol approved by your research ethics board. If you are unable to adhere to our open data policy, please kindly revise your statement to explain your reasoning and we will seek the editor's input on an exemption. Please be assured that, once you have provided your new statement, the assessment of your exemption will not hold up the peer review process.

6. In the online submission form you indicate that your data is not available for proprietary reasons and have provided a contact point for accessing this data. Please note that your current contact point is a co-author on this manuscript. According to our Data Policy, the contact point must not be an author on the manuscript and must be an institutional contact, ideally not an individual. Please revise your data statement to a non-author institutional point of contact, such as a data access or ethics committee, and send this to us via return email. Please also include contact information for the third party organization, and please include the full citation of where the data can be found.

7. If the reviewer comments include a recommendation to cite specific previously published works, please review and evaluate these publications to determine whether they are relevant and should be cited. There is no requirement to cite these works unless the editor has indicated otherwise.

[Note: HTML markup is below. Please do not edit.]

Reviewers' comments:

Reviewer's Responses to Questions

**Comments to the Author**

1. Is the manuscript technically sound, and do the data support the conclusions?

Reviewer #1: Partly

Reviewer #2: Yes

**********

2. Has the statistical analysis been performed appropriately and rigorously?

Reviewer #1: N/A

Reviewer #2: Yes

**********

3. Have the authors made all data underlying the findings in their manuscript fully available?

The PLOS Data policy

Reviewer #1: Yes

Reviewer #2: Yes

**********

4. Is the manuscript presented in an intelligible fashion and written in standard English?

Reviewer #1: Yes

Reviewer #2: Yes

**********

Reviewer #1: Editor-in-Chief

PLOS ONE

Subject: Reviewer recommendation for manuscript PONE-D-25-35293

Dear Editor

Thank you for considering me to review the manuscript entitled: "… The Referral Trajectories for Young People with Depression in low resource settings: A Qualitative study from Ghana and Zimbabwe (Manuscript ID: PONE-D-25-35293).

The study explores an important topic in mental health, particularly for population considered vulnerable with complexities of resources. This study is timely, for addressing metal health care gaps in the treatment of young people. However, several areas require revision to strengthen methodological rigor, clarity, and coherence before publication.

The following areas require the attention of the authors:

Title and abstract

- Title: ensure that the word “low resource” is correctly written. It should be ‘low-resource” instead of “low resource”.

- Abstract: there is grammar and spelling errors that require the attention on the authors e.g., line 10 refers to depressiom instead of depression. Additionally, the abstract lacks methodological details that justify the 102 participants e.g. how that sampled, and the data saturation or adequacy was decided on. Keywords are few- the thumb rule for keywords is 6-8.

Methods

- Design and theoretical framework: It is unclear how the phenomenological theory was used in the context of a clear exploratory descriptive study. a phenomenological study focuses on lived experiences while exploratory qualitative research explored a specific phenomenon without consideration of the lived experiences, therefore even the analytical approach for a clear exploratory descriptive study would differ from a phenomenological study. see line 116-121 vs line 12. The authors are advised to clearly write this section. Perceptions cannot be understood through lived experienced, there is a clear discrepancy that needs to be clarified see line 119-121- “Phenomenological research focuses on participants lived experiences of a phenomenon by understanding their perceptions, feelings, and thoughts about the phenomenon and the meaning they attach to such experiences”.

- Study context: it is not clear why the age varied in the different contexts even with the -current justification, there is a need to expand this section as it may have influenced comparability. If not, this should be expanded or detailed under limitations. See line 88-95.

- Participant recruitment: while the authors mention that the key informants were recommended, there is no mention of how they were approached and how informed consent was obtained.

- Number of participants: there is a discrepancy between the number of participants reported on the abstract vs the number of participants presented on Table 1 (102 vs 116), see line 13 vs line 142. It is recommended that the authors clarify the correct sample size.

- Data collection: while the manuscript reflects that an interview guide included a core list of open-ended questions. It does not reflect how they were formulated and pretested. Additionally, it does not reflect nature of questions asked. To correct this, please add the central question that was used, and a sample of the probing questions that were used since they were predetermined judging from the statement “The interview guides included a core list of open-ended questions and anticipated follow-up question” to interview. Translation in qualitative research is handled with careful considerations; how did the authors ensure that the meaning was maintained as there is no mention of backward translation?

- Data analysis: please manage the repeated headings under this section. There is no mention of the data analysis method used and the steps thereof. Ensure that this section is well described, include the method used to analyse the data, the role of the researchers during this process and describe if an independent qualitative data analyst was used , and how were the themes agreed upon?

- Trustworthiness- there is no section detailing how trustworthiness was ensured throughout the manuscript. This section is crucial in qualitative research. How did the authors ensure that the findings accurately reflect the perspectives of the participants?

Findings

- A thematic table would represent the results well, and further should who the themes and subthemes link. There figure 2 reflects trajectories of Ghana only, does it mean these are the same in Zimbabwe? The postulations made by the authors are worrisome for a qualitative study- what happens to bracketing our preconceived ideas particularly if the study is guided by the phenomenological framework? Postulating is therefore contradictory.

Discussion

- While the discussion is within a comparative approach, the discussion lacks sufficient contrast or integration of the findings from the two contexts. It is therefore important to be deliberate about this, there is a need for the authors to compare and contrast the two contexts in depth and highlight country specific (such as barriers and enablers) – see discussion section, page 21-25.

Limitations

- This section does not capture the potential bias introduced in this study by postulating. It is also necessary to expand this section to include how the lack of voice of the youth might have influenced the findings, hence the need for further research.

Language and grammar

- There is a need for language editing to address grammatical and typographical errors. E.g., Depressiom instead of depression; widder instead of wider, etc.

Reference

- Please ensure that all references cited are on the list. Also ensure consistency on the list of references to adhere to the journal’s required format.

Conclusion

Based on the above concerns, there are still areas that need to be addressed by the authors, I therefore recommend revision to strengthen methodological rigor and suitability for publication in PLOS ONE.

Reviewer #2: The manuscript is technically sound and analysis of data is relevant. It is appreciated that the article point out that the work does not include the side of the young people. it is suggested that the problem be specified, CAMHS be written out in full under keywords, complete the quotation " f in line 265, Ghana is missing from the first sentence under discussion. Cross check he reference list to ensure alignment and include references 38-45 in the text.

**********

what does this mean?). If published, this will include your full peer review and any attached files.

If you choose “no”, your identity will remain anonymous but your review may still be made public.

**Do you want your identity to be public for this peer review?**  For information about this choice, including consent withdrawal, please see our Privacy Policy

Reviewer #1: **Yes:**  Andile Glodin Mokoena-de Beer

Reviewer #2: **Yes:**  Hermine Iita

**********

[NOTE: If reviewer comments were submitted as an attachment file, they will be attached to this email and accessible via the submission site. Please log into your account, locate the manuscript record, and check for the action link "View Attachments". If this link does not appear, there are no attachment files.]

To ensure your figures meet our technical requirements, please review our figure guidelines: https://journals.plos.org/plosone/s/figures

You may also use PLOS’s free figure tool, NAAS, to help you prepare publication quality figures: https://journals.plos.org/plosone/s/figures#loc-tools-for-figure-preparation.

NAAS will assess whether your figures meet our technical requirements by comparing each figure against our figure specifications.

Attachment Submitted filename: Reviewers recommendations PONE-D-25-35293.pdf

# Author comment: Author response to Decision Letter 1

**DOI:** 10.1371/journal.pone.0358255.r002

25 Mar 2026

Reviewer 1

Title and abstract

- Title: ensure that the word “low resource” is correctly written. It should be ‘low-resource” instead of “low resource”.

A: Thank you for this correction. We have made the correction.

- Abstract: there is grammar and spelling errors that require the attention on the authors e.g., line 10 refers to depressiom instead of depression.

A: The spelling errors have been corrected. Thank you for the observation.

-Additionally, the abstract lacks methodological details that justify the 102 participants e.g. how that sampled, and the data saturation or adequacy was decided on.

A: Thank you for this observation. The methodology has been amended to include the relevant information which was initially omitted. (lines 12 to 18)

-Keywords are few- the thumb rule for keywords is 6-8.

A: We have made the necessary changes and have added more key words

Methods

- Design and theoretical framework: It is unclear how the phenomenological theory was used in the context of a clear exploratory descriptive study. a phenomenological study focuses on lived experiences while exploratory qualitative research explored a specific phenomenon without consideration of the lived experiences, therefore even the analytical approach for a clear exploratory descriptive study would differ from a phenomenological study. see line 116-121 vs line 12. The authors are advised to clearly write this section. Perceptions cannot be understood through lived experienced, there is a clear discrepancy that needs to be clarified see line 119-121- “Phenomenological research focuses on participants lived experiences of a phenomenon by understanding their perceptions, feelings, and thoughts about the phenomenon and the meaning they attach to such experiences”.

A: Thank you for pointing this out. True to what you have pointed out, this study does not entirely conform to the philosophical underpinnings of phenomenology. As such, we have removed the section of theoretical framework, and we have maintained what was initially mentioned in the abstract that this was an exploratory descriptive qualitative study. (line 100)

- Study context: it is not clear why the age varied in the different contexts even with the -current justification, there is a need to expand this section as it may have influenced comparability. If not, this should be expanded or detailed under limitations. See line 88-95.

A: You have raised an important issue which we are of the view that we attempted to explain in lines 105 to 110 that the difference in age groups between the two countries was necessitated by the distinct focus of each study setting i.e schools in Ghana and clinics in Zimbabwe. As explained in the article, in Ghana, for instance, schools were purposively chosen as study sites. For us to align with the school-going age range and the project’s efforts to strengthen existing school-based mental health systems, the age range of 15 to 18 was thus chosen. On the contrary, in Zimbabwe, the age range was a bit wide as the study focused on clinics where all young people patronise.

Additionally, we wish to emphasize that the goal of this study was not necessary to compare the two countries of Zimbabwe and Ghana because they are both classified low-resourced settings . As such, the two countries were observed as one entity, so to speak. We sincerely hope this explanation suffices, should there be need for more clarification please let us know.

- Participant recruitment: while the authors mention that the key informants were recommended, there is no mention of how they were approached and how informed consent was obtained.

A: We have now elaborated that the participants were approached telephonically or via email (line 141). Regarding informed consent, this information has been provided under ethical consideration section (lines 244 to 249)

- Number of participants: there is a discrepancy between the number of participants reported on the abstract vs the number of participants presented on Table 1 (102 vs 116), see line 13 vs line 142. It is recommended that the authors clarify the correct sample size.

A: Thank you for pointing out this discrepancy and we wish to inform you that the necessary changes have been made in both the abstract (lines 13 to 16) and in table 1 (line 162).

- Data collection: while the manuscript reflects that an interview guide included a core list of open-ended questions. It does not reflect how they were formulated and pretested. Additionally, it does not reflect nature of questions asked. To correct this, please add the central question that was used, and a sample of the probing questions that were used since they were predetermined judging from the statement “The interview guides included a core list of open-ended questions and anticipated follow-up question” to interview.

A: We have added one central question that was used for interviews involving nurses and one for young people (lines 193 to 198). Additionally, we have provided all the topic guides which were used (included in the Supporting Information S2 to S9).

Translation in qualitative research is handled with careful considerations; how did the authors ensure that the meaning was maintained as there is no mention of backward translation?

A: We concur with you that indeed translation is very crucial in qualitative research as it may lead to distortion of what was said by a participant if not properly done. To that effect, we wish to report that indeed back-translation was done by a different research assistant to ensure accuracy (lines 203 to 205). This has also been taken care of in the “Trustworthiness” section.

- Data analysis: please manage the repeated headings under this section.

A: We have deleted the repetition

There is no mention of the data analysis method used and the steps thereof. Ensure that this section is well described, include the method used to analyse the data, the role of the researchers during this process and describe if an independent qualitative data analyst was used, and how were the themes agreed upon?

A: Thank you for this question. It is indeed crucial for us to provide details regarding how data analysis was done including the steps that were followed. However, we are of the view that we provided all the necessary information regarding the data analysis (lines 210 to 227). Kindly enlighten us if this information is still not adequate and how we can go about it differently. We will really appreciate your insights.

- Trustworthiness- there is no section detailing how trustworthiness was ensured throughout the manuscript. This section is crucial in qualitative research. How did the authors ensure that the findings accurately reflect the perspectives of the participants?

A: Thank you for bringing this up. A section on trustworthiness has been added from lines 233 to 242.

Findings

- A thematic table would represent the results well, and further should who the themes and subthemes link.

A: We have included a table which shows both themes and subthemes (table 2)

-There figure 2 reflects trajectories of Ghana only, does it mean these are the same in Zimbabwe?

A: Figure 2 reflects trajectories of both countries of Ghana and Zimbabwe. We have thus amended the caption to reflect this. Thank you for your comment as it has reminded us to clarify this.

-The postulations made by the authors are worrisome for a qualitative study- what happens to bracketing our preconceived ideas particularly if the study is guided by the phenomenological framework? Postulating is therefore contradictory.

A: Thank you for making this important observation. Kindly note that we have removed the whole phrase that talked about postulating. We have subsequently amended the figure 1 by removing the arrows that reflected our postulations and have maintained our bracketing.

Discussion

- While the discussion is within a comparative approach, the discussion lacks sufficient contrast or integration of the findings from the two contexts. It is therefore important to be deliberate about this, there is a need for the authors to compare and contrast the two contexts in depth and highlight country specific (such as barriers and enablers) – see discussion section, page 21-25.

A: We have revised the discussion section and have attempted to make some comparison even though our main focus for the study was not necessarily to make a comparison of the two countries because they are both low-resource countries. (lines 638 to 558)

Limitations

- This section does not capture the potential bias introduced in this study by postulating.

A: Having removed the paragraph on postulation, we would like to believe that this is no longer an issue to be mentioned in the limitation. We hope that is in order.

-It is also necessary to expand this section to include how the lack of voice of the youth might have influenced the findings, hence the need for further research.

A: I am glad to report that we went back to our data, and we were able to find some relevant information from the young people on this topic, as such, we have finally included young people’s input into the study. To that effect, you may have noticed that we have included some direct quotes from the young people. This therefore implies that the demographic details of participants have subsequently changed.

Language and grammar

- There is a need for language editing to address grammatical and typographical errors. E.g., Depressiom instead of depression; widder instead of wider, etc.

A: Thank you for pointing out this. We have made the necessary corrections (lines 5, 10 & 514)

Reference

- Please ensure that all references cited are on the list. Also ensure consistency on the list of references to adhere to the journal’s required format.

A: Thank you for bringing this anomaly. We have revised the whole reference list and in-text citations using a software to ensure consistency.

Conclusion

Based on the above concerns, there are still areas that need to be addressed by the authors, I therefore recommend revision to strengthen methodological rigor and suitability for publication in PLOS ONE.

A: Thank you for taking your time to review our paper. We have tried to consider and address all the concerns raised. We are happy to clarify more if need be and we are also open for more comments and suggestions. We hope our revised paper meets PLOS ONE’s favourable consideration.

NB: we added a section on study implications, which we thought was an important omission worthy adding. (lines 691 to 70

Reviewer #2:

-The manuscript is technically sound and analysis of data is relevant.

A: Thank you for the encouraging words. We hope to improve the paper to make it more scientifically sound and readable.

-It is appreciated that the article point out that the work does not include the side of the young people. it is suggested that the problem be specified,

A: Thank you for bringing this up. Indeed, lack of young people’s voice was a big limitation. I am glad to report that we went back to our data, and we were able to find some relevant information from the young people on this topic, as such, we have finally included young people’s input into the study. To that effect, you may have noticed that we have included some direct quotes from the young people. This therefore implies that the demographic details of participants have subsequently changed.

CAMHS be written out in full under keywords,

A: Thank you for pointing this out. We have made the necessary changes (line 29)

complete the quotation " f in line 265,

A: We have corrected the quotation (line 348)

-Ghana is missing from the first sentence under discussion.

A: Thank you for pointing out this omission. We have added it on line 538.

-Cross check the reference list to ensure alignment and include references 38-45 in the text.

A: Thank you for bringing this anomaly. We have revised the whole reference list and in-text citations using a software to ensure consistency.

# Peer review: Decision Letter 1

**DOI:** 10.1371/journal.pone.0358255.r003

**Authors:** Dorothy Serwaa Boakye

19 Jun 2026

The referral trajectories for young people with depression in low-resource settings: A qualitative study from Ghana and Zimbabwe

PLOS One

Dear Dr. Makiyi,

Thank you for submitting your manuscript to PLOS ONE. After careful consideration, we feel that it has merit but does not fully meet PLOS ONE’s publication criteria as it currently stands. Therefore, we invite you to submit a revised version of the manuscript that addresses the points raised during the review process.

Please submit your revised manuscript by Aug 03 2026 11:59PM If you will need more time than this to complete your revisions, please reply to this message or contact the journal office at plosone@plos.org. When you're ready to submit your revision, log on to https://www.editorialmanager.com/pone/ and select the 'Submissions Needing Revision' folder to locate your manuscript file.

- A letter that responds to each point raised by the academic editor and reviewer(s). You should upload this letter as a separate file labeled 'Response to Reviewers'.
- A marked-up copy of your manuscript that highlights changes made to the original version. You should upload this as a separate file labeled 'Revised Manuscript with Track Changes'.
- An unmarked version of your revised paper without tracked changes. You should upload this as a separate file labeled 'Manuscript'.

If you would like to make changes to your financial disclosure, please include your updated statement in your cover letter. Guidelines for resubmitting your figure files are available below the reviewer comments at the end of this letter.

If applicable, we recommend that you deposit your laboratory protocols in protocols.io to enhance the reproducibility of your results. Protocols.io assigns your protocol its own identifier (DOI) so that it can be cited independently in the future. For instructions see: https://journals.plos.org/plosone/s/submission-guidelines#loc-laboratory-protocols. Additionally, PLOS ONE offers an option for publishing peer-reviewed Lab Protocol articles, which describe protocols hosted on protocols.io.

Read more information on sharing protocols at https://plos.org/protocols?utm_medium=editorial-email&utm_source=authorletters&utm_campaign=protocols.As the corresponding author, your ORCID iD is verified in the submission system and will appear in the published article. PLOS supports the use of ORCID, and we encourage all coauthors to register for an ORCID iD and use it as well. Please encourage your coauthors to verify their ORCID iD within the submission system before final acceptance, as unverified ORCID iDs will not appear in the published article. *Only*  the individual author can complete the verification step; PLOS staff *cannot*  verify ORCID iDs on behalf of authors.

We look forward to receiving your revised manuscript.

Kind regards,

Dorothy Serwaa Boakye, PGDE,MA,Mphil, RN, BSc

Academic Editor

PLOS One

Journal Requirements:

If the reviewer comments include a recommendation to cite specific previously published works, please review and evaluate these publications to determine whether they are relevant and should be cited. There is no requirement to cite these works unless the editor has indicated otherwise.

Please review your reference list to ensure that it is complete and correct. If you have cited papers that have been retracted, please include the rationale for doing so in the manuscript text, or remove these references and replace them with relevant current references. Any changes to the reference list should be mentioned in the rebuttal letter that accompanies your revised manuscript. If you need to cite a retracted article, indicate the article’s retracted status in the References list and also include a citation and full reference for the retraction notice.

Additional Editor Comments:

This thematic table 2 has a structural problem that requires your attention. As presented, it reads:

*(none listed)* </td></tr><tr><td class="border-b-0.5 border-[hsl(var(--border-300)/0.3)] py-2 pr-4 align-top">2. Systems factors — Young people's trajectories for specialized mental health services</td><td class="border-b-0.5 border-[hsl(var(--border-300)/0.3)] py-2 pr-4 align-top">*(none listed)*

The problem is **asymmetry and incompleteness** . Themes 1 and 2 have no sub-themes listed, while Theme 3 has two. This creates the impression that either:

- Themes 1 and 2 genuinely have no sub-themes (in which case, why present a themes-and-subthemes table?), or
- The authors forgot to populate those cells.

Reading the actual findings section, there *are*  identifiable sub-themes within Themes 1 and 2 that could have been named. For Theme 1, the text describes counselling services, screenings, school health programmes, and de-escalation — these could be organised as sub-themes. For Theme 2, there are at least two distinguishable components: the role of multiple first points of contact (peers, formal and informal agents), and the structural pathways within formal care (school ladder, gatekeeper system, parental involvement). The authors needed to have gone back to their data and named these sub-themes explicitly before constructing the table.

The table as structured does not fulfil the purpose a thematic table should serve in a qualitative paper, which is to give the reader a clear map of the analytical landscape before they enter the findings. I would recommend that you populate Themes 1 and 2 with properly named sub-themes that reflect the content of those sections.

[Note: HTML markup is below. Please do not edit.]

Reviewers' comments:

Reviewer's Responses to Questions

**Comments to the Author**

Reviewer #2: All comments have been addressed

Reviewer #3: All comments have been addressed

**********

2. Is the manuscript technically sound, and do the data support the conclusions?

Reviewer #2: Partly

Reviewer #3: Partly

**********

3. Has the statistical analysis been performed appropriately and rigorously?

Reviewer #2: N/A

Reviewer #3: Yes

**********

4. Have the authors made all data underlying the findings in their manuscript fully available?

The PLOS Data policy

Reviewer #2: No

Reviewer #3: Yes

**********

5. Is the manuscript presented in an intelligible fashion and written in standard English?

Reviewer #2: Yes

Reviewer #3: Yes

**********

Reviewer #2: Thank you for the adjustments made in response to the recommendations.

Kindly adjust (1) the technical aspect under keywords ( Youth,;).

(2) May the reviewer have access to the transcripts and recordings for confirmability check ?

Reviewer #3: Based on a review of the manuscript and the authors’ responses to the reviewers, here are a few areas that require further revision or careful proofreading to ensure consistency and methodological rigour.

1. Language and Qualitative Terminology

• Reviewer 1 explicitly stated that “postulations” are worrisome for a qualitative study. While your response states that the phrase was removed, the term “we postulate” still appears in the Discussion section (e.g., lines 543 and 588) regarding the impact of time spent with service providers. You should replace this with more appropriate qualitative phrasing, such as “the findings suggest” or “it is plausible that.”

• While you have addressed the “low-resource” hyphenation in the title, ensure that “school-going” and “low-resource” are consistently hyphenated throughout the entire body of the text.

2. Results and Figures

• Reviewer 1 noted that your postulations in Figure 1 were contradictory to the "bracketing" required in qualitative research. Ensure the final version of Figure 1 is strictly based on participants' reported trajectories and does not include the speculative arrows the reviewer flagged.

• The authors amended the caption of Figure 2 to reflect both countries. However, the text describing Figure 2 still largely focuses on “school-going adolescents in Ghana”. If the ladder truly reflects both countries, the text should integrate Zimbabwean data into this section to avoid a “Ghana-heavy” results section.

3. Discussion and Comparative Depth

• Currently, the Discussion remains somewhat compartmentalised, discussing general literature for Ghana and Zimbabwe separately. To strengthen this, you should more deliberately compare your own findings from Navrongo and Harare in the Discussion, rather than just referencing existing literature for each country.

• The authors mention the lack of trust between biomedical and traditional/faith healers. This could be further strengthened by discussing if there were any observed differences in this collaboration (or lack thereof) between the Ghanaian and Zimbabwean sites.

4. Technical and Formatting Errors

• There is a clear formatting error around line 594, where the citation appears as “(37)(37)”.

• Ensure the quotation mentioned (line 265) is fully closed and grammatically integrated into the surrounding text.

Thank you.

**********

what does this mean?). If published, this will include your full peer review and any attached files.

If you choose “no”, your identity will remain anonymous but your review may still be made public.

**Do you want your identity to be public for this peer review?**  For information about this choice, including consent withdrawal, please see our Privacy Policy

Reviewer #2: **Yes:**  Hermine Iita

Reviewer #3: No

**********

[NOTE: If reviewer comments were submitted as an attachment file, they will be attached to this email and accessible via the submission site. Please log into your account, locate the manuscript record, and check for the action link "View Attachments". If this link does not appear, there are no attachment files.]

To ensure your figures meet our technical requirements, please review our figure guidelines: https://journals.plos.org/plosone/s/figures

You may also use PLOS’s free figure tool, NAAS, to help you prepare publication quality figures: https://journals.plos.org/plosone/s/figures#loc-tools-for-figure-preparation.

NAAS will assess whether your figures meet our technical requirements by comparing each figure against our figure specifications.

# Author comment: Author response to Decision Letter 2

**DOI:** 10.1371/journal.pone.0358255.r004

28 Jul 2026

Additional Editor Comments:

This thematic table 2 has a structural problem that requires your attention. As presented, it reads:

Theme Sub-Theme

1. Supply factors — Availability and nature of specialised mental healthcare and services (none listed)

2. Systems factors — Young people's trajectories for specialized mental health services (none listed)

3. Navigating the referral pathways — Referral barriers and enablers a. Existing referral barriers / b. Existing referral enablers

The problem is asymmetry and incompleteness. Themes 1 and 2 have no sub-themes listed, while Theme 3 has two. This creates the impression that either:

• Themes 1 and 2 genuinely have no sub-themes (in which case, why present a themes-and-subthemes table?), or

• The authors forgot to populate those cells.

Reading the actual findings section, there are identifiable sub-themes within Themes 1 and 2 that could have been named. For Theme 1, the text describes counselling services, screenings, school health programmes, and de-escalation — these could be organised as sub-themes. For Theme 2, there are at least two distinguishable components: the role of multiple first points of contact (peers, formal and informal agents), and the structural pathways within formal care (school ladder, gatekeeper system, parental involvement). The authors needed to have gone back to their data and named these sub-themes explicitly before constructing the table.

The table as structured does not fulfil the purpose a thematic table should serve in a qualitative paper, which is to give the reader a clear map of the analytical landscape before they enter the findings. I would recommend that you populate Themes 1 and 2 with properly named sub-themes that reflect the content of those sections.

R: Thank you for pointing this out. We have revised both the table 2 (thematic table) as well as the subsequent text within the findings section to reflect the changes in the table. Suffice to mention that we went back to the data, as such, we have added a few content in the result section and where necessary, we have also moved some texts to reflect the new subthemes that have been created after a thorough reading of the initial text and the data.

Reviewers' comments:

Reviewer's Responses to Questions

Comments to the Author

1. If the authors have adequately addressed your comments raised in a previous round of review and you feel that this manuscript is now acceptable for publication, you may indicate that here to bypass the “Comments to the Author” section, enter your conflict of interest statement in the “Confidential to Editor” section, and submit your "Accept" recommendation.

Reviewer #2: All comments have been addressed

Reviewer #3: All comments have been addressed

________________________________________

2. Is the manuscript technically sound, and do the data support the conclusions?

The manuscript must describe a technically sound piece of scientific research with data that supports the conclusions. Experiments must have been conducted rigorously, with appropriate controls, replication, and sample sizes. The conclusions must be drawn appropriately based on the data presented.

Reviewer #2: Partly

Reviewer #3: Partly

________________________________________

3. Has the statistical analysis been performed appropriately and rigorously?

Reviewer #2: N/A

Reviewer #3: Yes

________________________________________

4. Have the authors made all data underlying the findings in their manuscript fully available?

The PLOS Data policy requires authors to make all data underlying the findings described in their manuscript fully available without restriction, with rare exception (please refer to the Data Availability Statement in the manuscript PDF file). The data should be provided as part of the manuscript or its supporting information, or deposited to a public repository. For example, in addition to summary statistics, the data points behind means, medians and variance measures should be available. If there are restrictions on publicly sharing data—e.g. participant privacy or use of data from a third party—those must be specified.

Reviewer #2: No

Reviewer #3: Yes

________________________________________

5. Is the manuscript presented in an intelligible fashion and written in standard English?

PLOS ONE does not copyedit accepted manuscripts, so the language in submitted articles must be clear, correct, and unambiguous. Any typographical or grammatical errors should be corrected at revision, so please note any specific errors here.

Reviewer #2: Yes

Reviewer #3: Yes

________________________________________

6. Review Comments to the Author

Please use the space provided to explain your answers to the questions above. You may also include additional comments for the author, including concerns about dual publication, research ethics, or publication ethics. (Please upload your review as an attachment if it exceeds 20,000 characters)

Reviewer #2: Thank you for the adjustments made in response to the recommendations.

Kindly adjust (1) the technical aspect under keywords ( Youth,;).

R: Done

(2) May the reviewer have access to the transcripts and recordings for confirmability check?

R: Thank you for expressing interest to access the transcripts and recordings for confirmability check. We appreciate that interest and under normal circumstances, we would have been glad to share. However, as mentioned in the Data availability Statement: “The data underlying the results presented in this study are available from the Institute of Psychiatry, Psychology & Neuroscience at King’s College London. Data access requests should be directed to research.data@kcl.ac.uk.

Reviewer #3: Based on a review of the manuscript and the authors’ responses to the reviewers, here are a few areas that require further revision or careful proofreading to ensure consistency and methodological rigour:

1. Language and Qualitative Terminology

• Reviewer 1 explicitly stated that “postulations” are worrisome for a qualitative study. While your response states that the phrase was removed, the term “we postulate” still appears in the Discussion section (e.g., lines 543 and 588) regarding the impact of time spent with service providers. You should replace this with more appropriate qualitative phrasing, such as “the findings suggest” or “it is plausible that.”

R: Thank you for this observation. We have indeed made the necessary revision (line 620)

• While you have addressed the “low-resource” hyphenation in the title, ensure that “school-going” and “low-resource” are consistently hyphenated throughout the entire body of the text.

R: We have checked and have made sure that we are consistent with the use of hyphen in all compound adjectives such as “low-cadre healthcare workers” (line 646)

2. Results and Figures

• Reviewer 1 noted that your postulations in Figure 1 were contradictory to the "bracketing" required in qualitative research. Ensure the final version of Figure 1 is strictly based on participants' reported trajectories and does not include the speculative arrows the reviewer flagged.

R: Thank you. This was corrected in the initial revision, and it reflects what the participants said and not our postulations.

• The authors amended the caption of Figure 2 to reflect both countries. However, the text describing Figure 2 still largely focuses on “school-going adolescents in Ghana”. If the ladder truly reflects both countries, the text should integrate Zimbabwean data into this section to avoid a “Ghana-heavy” results section.

R: Thank you for this observation. The figure 2 illustrates the trajectories for School-going adolescents from both countries and not just Ghana. The preceding text now has additional data from Zimbabwe to strike a balance. Additionally, figure 1 illustrates the trajectories for both countries as well.

3. Discussion and Comparative Depth

• Currently, the Discussion remains somewhat compartmentalised, discussing general literature for Ghana and Zimbabwe separately. To strengthen this, you should more deliberately compare your own findings from Navrongo and Harare in the Discussion, rather than just referencing existing literature for each country.

R: We appreciate your observation regarding this. We have tried to make comparisons between Ghana and Zimbabwe where necessary. Suffice to mention that most of the findings from the two countries were similar and where there were any differences, they were minor for instance on barriers, and this was pointed out in the discussion. We have thus ended the discussion section by noting that “our study demonstrates that young people in Ghana and Zimbabwe navigate remarkably similar pathways to specialist mental healthcare, shaped by the same constellation of FPOC, barriers, gatekeeping structures, and enablers”.

• The authors mention the lack of trust between biomedical and traditional/faith healers. This could be further strengthened by discussing if there were any observed differences in this collaboration (or lack thereof) between the Ghanaian and Zimbabwean sites.

R: Thank you for bringing this out. We have mentioned in our discussion that there is some level of networking and collaboration among formal players like biomedical healthcare workers and key players like the police, teachers, social workers etc. We went on to mention that “In both Ghana and Zimbabwe, the relationship between the biomedical/formal healthcare providers and the informal providers like traditional- / faith healers did not come out clearly from our data” ….The “lack of trust” is something that was found in other studies and we raised it as part of our argument that there are such barriers that impede collaboration between the formal and informal players.

4. Technical and Formatting Errors

• There is a clear formatting error around line 594, where the citation appears as “(37)(37)”.

R: We have corrected this error by deleting it as it was misplaced.

• Ensure the quotation mentioned (line 265) is fully closed and grammatically integrated into the surrounding text.

R: Done (lines 259 to 261)

Thank you.

# Peer review: Decision Letter 2

**DOI:** 10.1371/journal.pone.0358255.r005

**Authors:** Dorothy Serwaa Boakye

31 Aug 2026

The referral trajectories for young people with depression in low-resource settings: A qualitative study from Ghana and Zimbabwe

PONE-D-25-35293R2

Dear Dr. Timothy Makiyi,

We’re pleased to inform you that your manuscript has been judged scientifically suitable for publication and will be formally accepted for publication once it meets all outstanding technical requirements.

Within one week, you’ll receive an e-mail detailing the required amendments. When these have been addressed, you’ll receive a formal acceptance letter and your manuscript will be scheduled for publication.

An invoice will be generated when your article is formally accepted. Please note, if your institution has a publishing partnership with PLOS and your article meets the relevant criteria, all or part of your publication costs will be covered. Please make sure your user information is up-to-date by logging into Editorial Manager at Editorial Manager® and clicking the ‘Update My Information' link at the top of the page. For questions related to billing, please contact billing support.

If your institution or institutions have a press office, please notify them about your upcoming paper to help maximize its impact. If they’ll be preparing press materials, please inform our press team as soon as possible -- no later than 48 hours after receiving the formal acceptance. Your manuscript will remain under strict press embargo until 2 pm Eastern Time on the date of publication. For more information, please contact onepress@plos.org.

Kind regards,

Dorothy Serwaa Boakye, PGDE,MA,Mphil, RN, BSc

Academic Editor

PLOS One

Additional Editor Comments (optional):

Reviewers' comments:

Reviewer's Responses to Questions

**Comments to the Author**

Reviewer #3: All comments have been addressed

Reviewer #4: All comments have been addressed

**********

2. Is the manuscript technically sound, and do the data support the conclusions?

Reviewer #3: No

Reviewer #4: Yes

**********

3. Has the statistical analysis been performed appropriately and rigorously?

Reviewer #3: Yes

Reviewer #4: N/A

**********

4. Have the authors made all data underlying the findings in their manuscript fully available?

The PLOS Data policy

Reviewer #3: Yes

Reviewer #4: Yes

**********

5. Is the manuscript presented in an intelligible fashion and written in standard English?

Reviewer #3: Yes

Reviewer #4: Yes

**********

Reviewer #3: Line 194: There is a typographical error with a double opening parenthesis “((”. Please correct this for clarity and grammatical accuracy.

Line 360: Please provide the full meaning of the abbreviation CHPS at first mention. Given the international readership of the journal, the abbreviation may not be familiar to all readers.

Lines 706–707: The phrase “as mentioned” appears to be repeated. Please revise the sentence to avoid unnecessary repetition and improve the readability of the text.

Reviewer #4: All comments by the reviewer have been addressed by the authors. Congratulations to the authors for the effort put in the revision of the paper

**********

what does this mean?). If published, this will include your full peer review and any attached files.

If you choose “no”, your identity will remain anonymous but your review may still be made public.

**Do you want your identity to be public for this peer review?**  For information about this choice, including consent withdrawal, please see our Privacy Policy

Reviewer #3: No

Reviewer #4: No

**********

# Editor report: Acceptance letter

**DOI:** 10.1371/journal.pone.0358255.r006

**Authors:** Dorothy Serwaa Boakye

PONE-D-25-35293R2

PLOS One

Dear Dr. Makiyi,

I'm pleased to inform you that your manuscript has been deemed suitable for publication in PLOS One. Congratulations! Your manuscript is now being handed over to our production team.

At this stage, our production department will prepare your paper for publication. This includes ensuring the following:

* All references, tables, and figures are properly cited

* All relevant supporting information is included in the manuscript submission,

* There are no issues that prevent the paper from being properly typeset

You will receive further instructions from the production team, including instructions on how to review your proof when it is ready. Please keep in mind that we are working through a large volume of accepted articles, so please give us a few days to review your paper and let you know the next and final steps.

Lastly, if your institution or institutions have a press office, please let them know about your upcoming paper now to help maximize its impact. If they'll be preparing press materials, please inform our press team within the next 48 hours. Your manuscript will remain under strict press embargo until 2 pm Eastern Time on the date of publication. For more information, please contact onepress@plos.org.

You will receive an invoice from PLOS for your publication fee after your manuscript has reached the completed accept phase. If you receive an email requesting payment before acceptance or for any other service, this may be a phishing scheme. Learn how to identify phishing emails and protect your accounts at https://explore.plos.org/phishing.

If we can help with anything else, please email us at customercare@plos.org.

Thank you for submitting your work to PLOS One and supporting open access.

Kind regards,

PLOS One Editorial Office Staff

on behalf of

Dr. Dorothy Serwaa Boakye

Academic Editor

PLOS One
